# Comprehensive molecular analysis of arginase-encoding genes in common wheat and its progenitor species

**DOI:** 10.1038/s41598-017-07084-0

**Published:** 2017-07-26

**Authors:** Maoyun She, Jing Wang, Xinmin Wang, Guixiang Yin, Ke Wang, Lipu Du, Xingguo Ye

**Affiliations:** 1 National Key Facility of Crop Gene Resources and Genetic Improvement/Institute of Crop Sciences, Chinese Academy of Agricultural Sciences, Beijing, 100081 P.R. China; 2 Crop Research Institute, Anhui Academy of Agricultural Sciences, Hefei, 230031 P.R. China

## Abstract

Arginase (ARG) contributes to nitrogen remobilization by conversion of arginine to ornithine and urea. However, wheat *ARG* genes have not yet been identified. Here we isolated and characterized *ARG* genes from wheat and its progenitor species and found that a single copy was present in wheat progenitors. Three common wheat *ARG* genes of *TaARG-2AS*, *TaARG-2BS*, and *TaARG-2DS* were experimentally assigned to the short arms of the group 2 chromosomes. We found an in-frame stop codon in *TaARG*-*2AS*, but not in the other two genes. The highest expression was detected in stems and sheaths for *TaARG-2BS* and in leaves for *TaARG*-*2DS*. Both genes have similar expression trend in different developmental stages, peaking at booting and grain filling stages. *TaARG*-*2BS* transcript was induced by high salinity and drought, whereas *TaARG*-*2DS* was induced by drought only, but neither of them were induced by low temperature. In addition, both genes showed analogous expression pattern upon powdery mildew (PM) infection in the resistant line Pm97033, with *TaARG-2BS* induced greatly at 72 h post PM infection. In contrast, no obvious transcripts were accumulated for *TaARG-2DS* in the PM susceptible line Wan7107. Monocot ARGs have more conserved mitochondrion-targeting signals and are more evolutionarily conserved than dicot ARGs.

## Introduction

Nitrogen (N) is an important element found in a variety of plant biological macromolecules and is the primary factor limiting plant growth and yield improvement^1^. Arginine (Arg), an N-rich amino acid, makes up 50% of the N content of total seed protein and is a primary component of the plant free amino acid pool, serving as a pivotal donor of substrates for glutamate and polyamine (PA) biosynthesis reactions. When needed, Arg can be catalyzed by arginase [*L*-Arg ureahydrolase (ARG) or amidinohydrolyase (ARGAH), EC 3.5.3.1] to produce ornithine (Orn) and urea, by nitric oxide synthase (NOS, EC 1.14.13.39) to be involved in NO-mediated regulatory pathway, and by arginine decarboxylase (ADC, EC 4.1.1.19) to modulate PA biosynthesis^2^. Among these enzymes, ARG is thought to play a key role in N remobilization and utilization^3^.

ARG is widely distributed in the biosphere and has evolved to catalyze the formation of different-sized polymers (ranging from monomers to octamers), which in turn has led to functional diversity in polymers^4^. Thus, more in-depth investigation is essential for unraveling *ARG* gene functions. Ever since the identification of the first *ARG* gene (*ARGAH1*) from *Arabidopsis thaliana* ^5^, many ARG-encoding genes have been subsequently isolated and characterized in plants. In tomato (*Lycopersicon esculentum* L.), there are two *ARG*s (*LeARG1*, *LeARG2*) in the genome^6^. In addition to having divergent protein coding sequences, these genes also have different expression patterns. *LeARG1* is preferentially expressed in roots; however, *LeARG2* is up-regulated by wounding and methyl jasmonate (MJ) treatment, indicating that the two *LeARGs* have different functions. In soybean (*Glycine max* L.), Goldraij and Polacco identified an *ARG* transcript, *AG1*, in seedlings. No transcript was detected in developing embryos, but at 3 to 5 d after seed germination there was high ARG activity. Southern blotting analysis suggested that *AG1* belongs to a small gene family in soybean that encodes proteins with deduced molecular weights ranging from 36.5 to 38.6 kDa^7^. Todd *et al*.^8^ isolated a cDNA encoding a 341 amino acid ARG homolog from a loblolly pine (*Pinus taeda* L.) cDNA library. This gene is found in single copy based on southern blotting. In rice (*Oryza sativa* L.), *OsARG* was successfully isolated through map-based cloning^9^. *OsARG* is a single-copy 6-exon gene coding for a protein of 340 amino acids and is constitutively expressed at all stages of rice development. So far, wheat *ARG* genes have not been characterized despite the availability of a wheat whole-genome sequence.

Considering that Arg and Orn are both precursors for the biosynthesis of PAs and that PAs confer plant tolerance to abiotic stresses, *ARG* is thought to be involved in plant responses to abiotic stresses^10–12^. Consistent with this hypothesis, chilling can induce the expression of *LeARG1* and *LeARG2* and increase ARG enzyme activity. Exogenous Arg treatment can alleviate fruit chilling injury and reduce electrolyte leakage, malondialdehyde content and peroxidase activity^13^. Conversely, there is enhanced tolerance to multiple abiotic stresses in knockout mutants of *Arabidopsis AtARGAH*s due to an increase in the direct downstream products of Arg catabolism, including PAs and NO^14^. The contradictory roles of *ARG* in stress response in tomato and *Arabidopsis* are due to the fact that three Arg catabolic pathways compete with each other. Once the ARG pathway is blocked, the production of PAs by the NOS and ADC pathways increases, contributing to abiotic stress tolerance^15, 16^. Far less is known about the relationship between *ARG* expression and abiotic stress in wheat. Manipulation of the expression of wheat *ARG* genes at the transcriptional level is important to dissect the relationship between ARG activity and wheat tolerance to abiotic and biotic stresses, considering that previous research has demonstrated the involvement of *ARG* overexpression in the resistance to biotic stresses^17, 18^. Brauc *et al*.^19^ observed that accumulation of *ARGAH2* mRNA was induced in *Arabidopsis* after inoculation with the necrotrophic pathogen *Botrytis cinerea*. Furthermore, ARG contributes to the conversion of *α*-difluoromethylarginine (DFMA) to *α*-difluoromethylornithine (DFMO), resulting in effective inhibition of the development of *Puccinia recondita* (leaf rust), *P. graminis* f. sp. *tritici* (stem rust), and *Erysiphe graminis* (powdery mildew, PM) in wheat^17^. Although wheat *ARG* has also been reported to contribute to the resistance to *P. striiformis* f. sp. *tritici* (PST, stripe rust)^18^, which *ARG* genes are the most important for the resistance is not yet clear because like most wheat genes, there are likely multiple *ARG* genes in wheat genome. In addition, Dubcovsky and Dvorak^20^ have pointed out that the genes found on the A, B, and D genomes are so similar to each other, only dominant mutations will lead to a distinguishing phenotype, which makes it even more important to determine which gene has the largest contribution to a given phenotype.

In this study, our objective was to identify wheat candidate genes accounting for N remobilization and responsive to stresses. To do this, we isolated *ARG* sequences from diploid and hexaploid wheat accessions. We then characterized the common wheat *ARG* genes and ascertained their copy number, chromosomal locations, and expression profiles in different tissues, at different developmental stages, and in response to various stresses. Our results will provide new insights into the molecular structures, phylogenetic relationships, and functional divergence of the *ARG* gene family in wheat.

## Results

### Isolation of *ARG* genes from common wheat and its progenitor species

**Figure 1:**
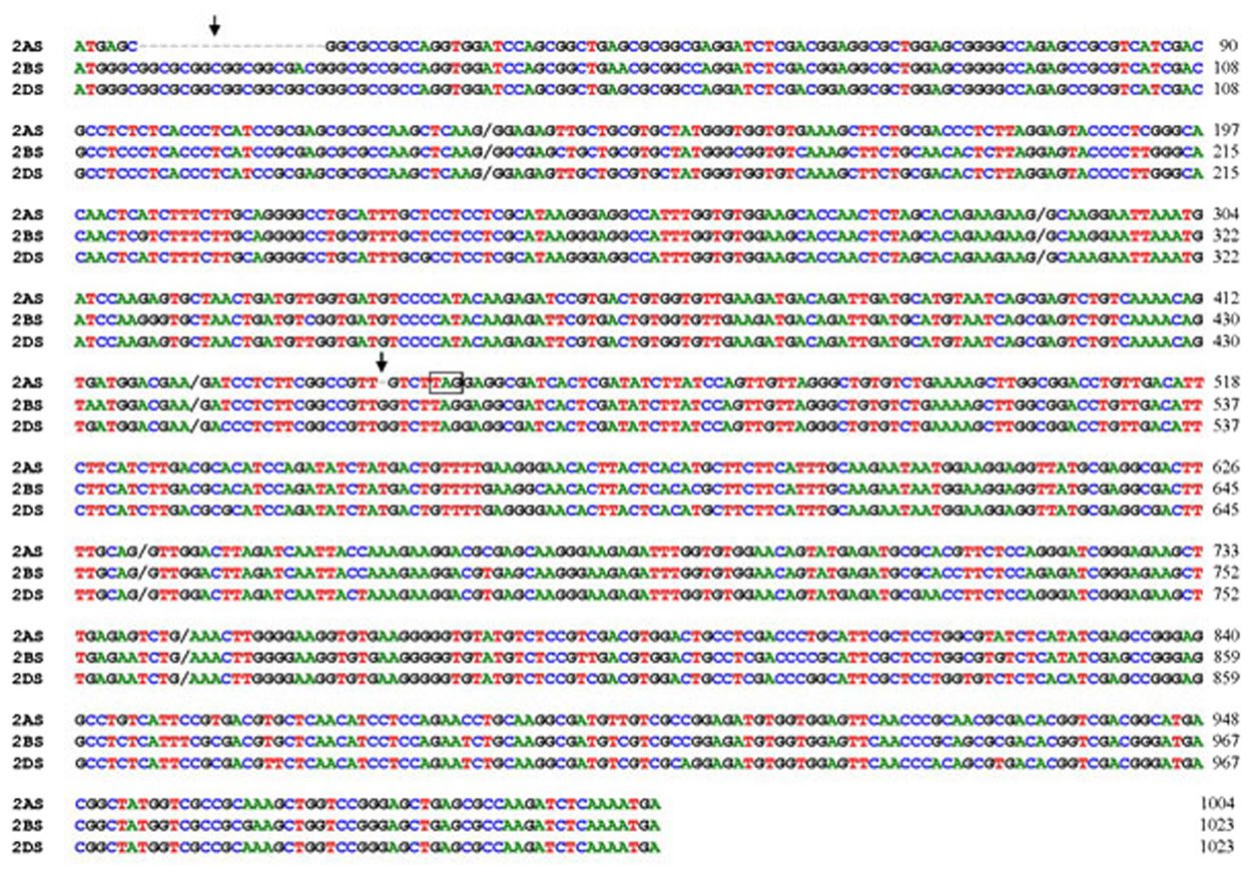
Sequence alignment of *ARG* cDNAs from the hexaploid wheat accession CB037. 2AS, 2BS, and 2DS represent chromosome names. A slash indicates the intron insertion site. Arrows show the deletions in *TaARG* located on chromosome 2AS that are not present in the *TaARG* genes on chromosomes 2BS and 2DS. The in-frame stop codon is shown in the box.

Using the OsARG amino acid sequence (LOCOs04g01590.1) as a query, a tBLASTn search was performed against the wheat tentative contig (TC) database (http://compbio.dfci.harvard.edu/tgi/). Two wheat TCs with high similarity to OsARG were retrieved. One sequence, TC409299, has many ambiguous bases (N) and was not included in subsequent analysis. The second sequence, TC381190, has two alternatively spliced forms. Next, a BLASTn search was conducted against the wheat expressed sequence tag (EST) database in NCBI to identify the correct TC381190 sequence. Three ESTs (GenBank accession Nos: CJ521864, CJ630469, CV767966) were found to encompass TC381190. These sequences contain two in-frame stop codons, TGA and TAG, located 120 and 114 nucleotides upstream of the start codon ATG, respectively, indicating that TC381190 encodes a full-length protein according to the criteria outlined by Min *et al*.^21^. To determine the number of *ARG* alleles in the common wheat genome, contigs with high sequence identity (E value = 0) were retrieved from the IWGSC database (International Wheat Genome Sequencing Consortium, http://www.wheatgenome.org/). Three contigs containing the entire *ARG* ORF were identified. Based on the wheat survey chromosome database, two contigs, TGACv1scaffold641144U and TGACv1scaffold643294U, were assigned to the short arms of chromosomes 2A and 2B, respectively, and TGACv1scaffold178572_2DS was assigned to chromosome 2DS. Gene-specific primer pairs were designed to flank the predicted ORFs (ORF Finder in NCBI, http://www.ncbi.nlm.nih.gov/gorf/gorf.html) (Table 1). Using PCR, cDNA/gDNA fragments for the three genes were amplified from the common wheat accession CB037. These fragments had lengths of 1053/3035, 1069/3079, and 1071/3873 bp. The exon-intron boundaries were determined by comparing the full-length cDNA sequence with the corresponding gDNA sequence. These boundaries have the classical intron splice boundary sequence “…GT…AG…”, and the three genes share high identity at the cDNA level and have the same 6-exon gene structure (Fig. 1). Based on the predicted chromosome locations, the three genes were designated as *TaARG-2AS*, *TaARG-2BS*, and *TaARG-2DS*. Further alignments with the rice OsARG amino acid sequence (GenBank accession number: HM369061) showed that OsARG has higher similarity to TaARG-2BS (92.1%) than to TaARG-2AS (45.6%) and TaARG-2DS (91.5%).

**Table 1:** Sequences of primer pairs used in this study.

Gene name	Forward primer (5′ → 3′)	Reverse primer (5′ → 3′)	Fragment size (bp)#	Annotation
*TaARG*	CTCGGCGATTCCTCGGCGATG^1^	TCTTTGCTGCTTCTCTACTGACT	3035/1053	Specific to chromosome 2AS
	CTCGACGATTCCTCGGCGATG^2^	CTGCTGCCTCTGTCCTGACTGT	3079/1069	Specific to chromosome 2BS
	CTCGACGATTCCTCGGCGATG^2^	CTTTGCTGCTTCGGTCCTGACT^3^	3873/1071	Specific to chromosome 2DS
*TmARG*	CTCGACGATTCCTCGGCGATG^2^	TCTTTGTTGATTCTGTCCTGACT	3284/1072	Specific to AA genome
*AelARG*/*AesARG*	CTCGGCGATTCCTCGGCGATG^1^	TTGCTGCCTCTGTCCTGACCGT	3206/1070	Specific to SS genome
*AetARG*	CTCGACGATTCCTCGGCGATG^2^	CTTTGCTGCTTCGGTCCTGACT^3^	3939/1071	Specific to DD genome
*TaARG-2AS*	TTTCCTATGATCGCACTCTTCC	GACGATGTTCTCATCGACTACGA	716	Chromosomal location
*TaARG-2BS*	CCTGTACTCCTTCAGTCGAATA	CTCAAGTCCTGCAGATACCATAC	690	Chromosomal location
*TaARG-2DS*	CTTTGGTGGAATGCTTCATCAG	GGGAGAGCAGTATAAGGAACA	703	Chromosomal location
*TaARG*	TTGGGCACAACTCATCTTTCTT	AGAAGGTTCGCATCTCATACTG	525	Probe amplification
*TaARG-2BS*	CGCATTCGCTCCTGGCGTGT	CGTCGACCGTGTCGCGCTGC	140	qRT-PCR
*TaARG-2DS*	GGCATTCGCTCCTGGTGTC	CGTCGACCGTGTCACGCTGT	140	qRT-PCR
Actin*	CTGACTGAGGCCCCTCTCAAC	CAAGGTCCAAACGAAGGATA	229	qRT-PCR

Primer sequences labeled with the same number have the same sequence.

#The numbers before and after the slashes represent the lengths of the gDNA and cDNA sequences, respectively.

*GenBank Accession number: AB181991.

**Figure 2:**
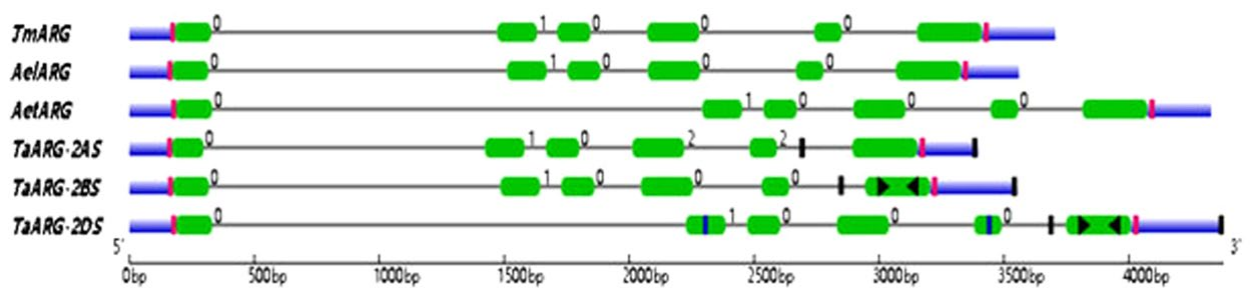
Structure of *ARG* genes in common wheat and wild diploid species. Tm, Ael, Aet, and Ta indicate *Triticum monococcum*, *Aegilops longissima*, *Aegilops tauschii*, and *Triticum aestivum*, respectively. Black lines represent introns, green rectangles represent exons, and blue rectangles represent UTRs. The columns mark the primer pairs used for the isolation of full-length *TaARG* genes (red), amplification of probe sequence for the southern blotting assay (yellow), and determination of chromosomal location (black). Primer pairs used for qRT-PCR analysis are indicated by horizontal triangles pointing in opposite directions. The number on the top left of the intron denotes intron phase.

Intriguingly, *TaARG*-*2AS* is a pseudogene due to an in-frame stop codon caused by two deletions (indicated by arrows in Fig. 1), which partially accounts for the low similarity between TaARG-2AS and OsARG. The early termination in TaARG-2AS was further confirmed by a contig named Traes2ASAD125CB18 in the gramene database (http://www.gramene.org/). To further verify the premature stop codon in *TaARG*-*2AS*, gene-specific primers were designed to obtain cDNA sequences from 50 selected common wheat accessions (Additional file 1). All sequences had the early stop codon. Next, we asked whether the early termination is common to wild diploid species. *ARG* genes were isolated from eighteen wild diploid species. All *ARG* genes in common wheat and wild diploid species have similar structures and the same intron phase except for *TaARG*-*2AS* (Fig. 2). And the full-length cDNAs of *ARG* genes from the wild species of AA genomes consistently have SNPs but no early termination codons. In addition, pairwise alignments were also performed for *ARG* genes from common wheat except that on chromosome 2AS and three diploid progenitors. The pairwise similarity between amino acid sequences was over 98%, and gDNA sequence identity ranged from 74.5 to 94.1%.

**Figure 3:**
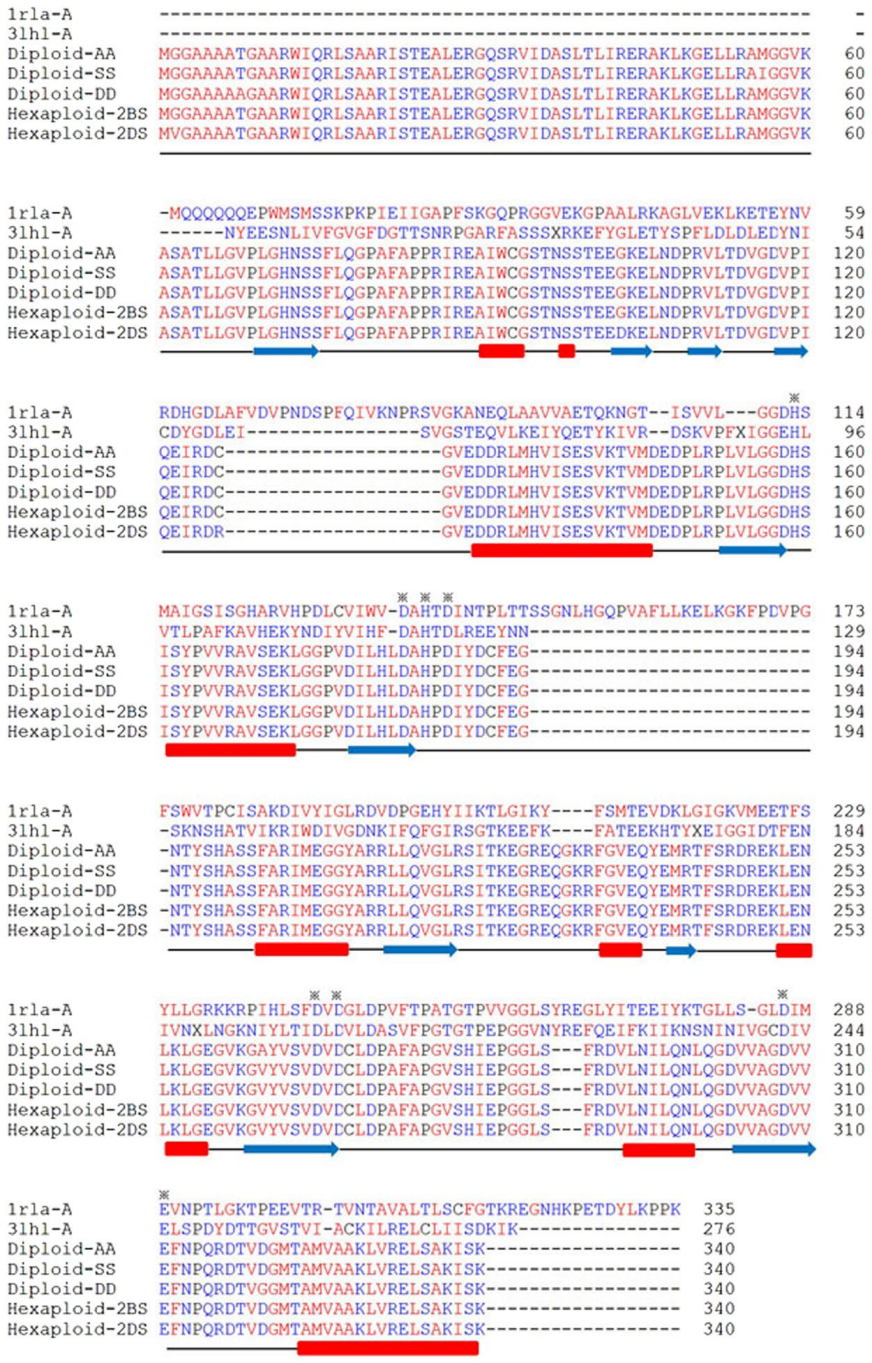
Alignment of ARG amino acid sequences from common wheat and its progenitor species. *α*-helixes and *β*-sheets are indicated by red rectangles and blue arrows, respectively. The asterisks indicate the functional conserved residues. The polar amino acid residues are shown in blue, and the remaining amino acids are shown in red.

To determine the protein structure, the tertiary structures of the ARG proteins in diploid and hexaploid wheat species were predicted based on homologous modeling. A crystal structure of agmatinase (PDB accession ID in SWISS MODEL: 3pzlB, GenBank accession ID: EQG02306), which had the lowest E-value of 3e-25, was obtained using the online template search tool in the ExPASy website (http://www.expasy.org). In total, ARG contains ten *β*-sheets and ten *α*-helixes (Fig. 3). In addition, according to the functional annotation of the type I ARG in rat (PDB accession ID in SWISS MODEL: 1rlaA, GenBank accession ID: EDL87779)^22^, the eight functional residues, including H159, D183, H185, D187, D268, D270, D308, and E311, are conserved in all ARGs from common wheat and diploid wild species. There are some sense mutations in homologous genes located on chromosomes 2BS and 2DS in different hexaploid wheat accessions (data not shown), which together with phenotype data may be regarded as useful markers for screening wheat accessions with dominant genes.

### Confirmation of the copy number and chromosomal localization of *TaARG* genes

**Figure 4:**
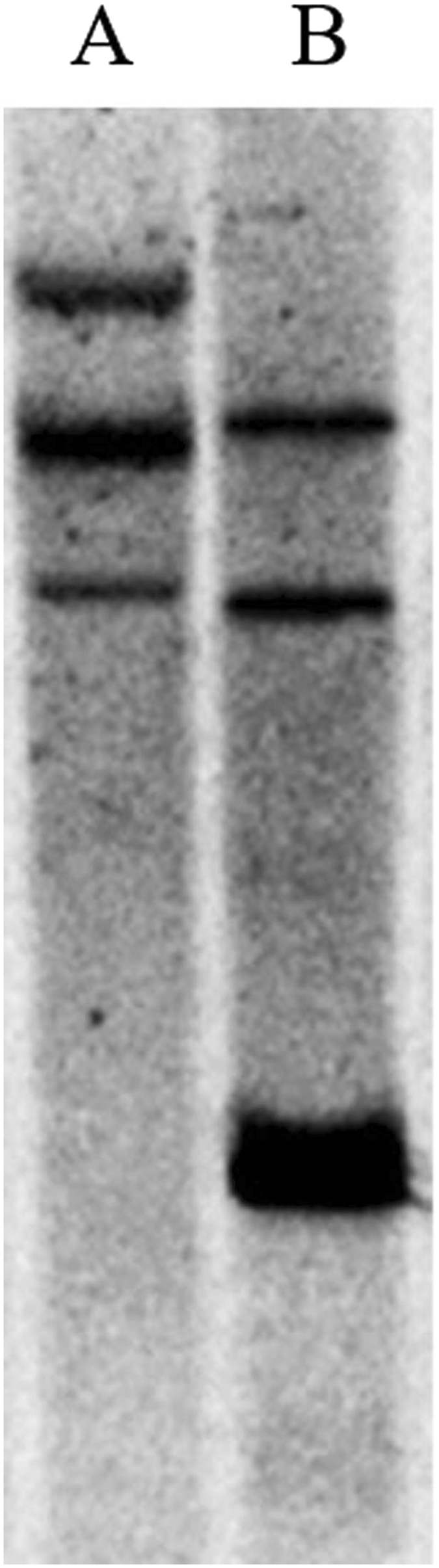
Southern blot analysis to determine *TaARG* copy number in the common wheat genome. (**A**) *Bam*HI-digested genomic DNA of CB037; (**B**) *Kpn*I-digested genomic DNA of CB037. acquisition tools and image processing software package. The photo was acquired by Tanon 5200 software (YPH-bio, Co. Ltd. Beijing, China).

**Figure 5:**
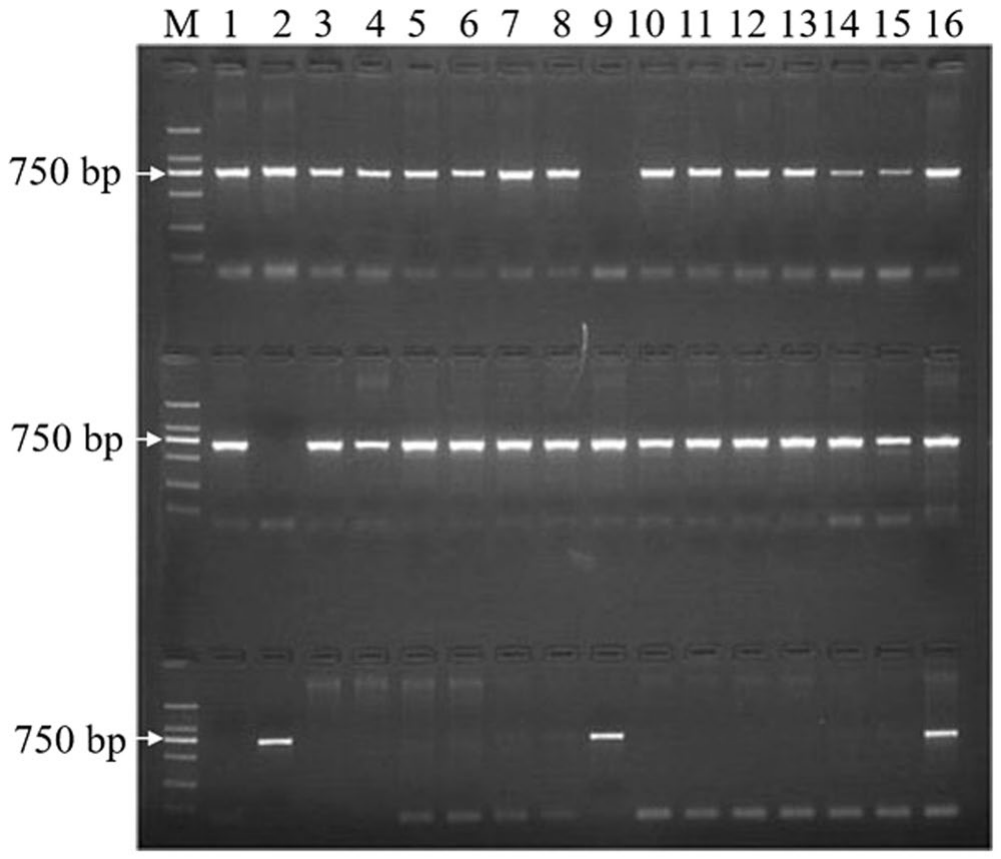
Determination of the chromosome location of *TaARG* genes using Langdon–Chinese Spring chromosome substitution lines. M: DL2000 DNA ladder (TianGen Biotech (Beijing) Co., Ltd.). The locations of *TaARG*s on chromosomes 2AS, 2BS, and 2DS, are shown in the top, middle, and bottom panels, respectively. Lanes 1–16: LDN-1D(1B), LDN-2D(2B), LDN-3D(3B), LDN-4D(4B), LDN-5D(5B), LDN-6D(6B), LDN-7D(7B), LDN-1D(1 A), LDN-2D(2 A), LDN-3D(3 A), LDN-4D(4 A), LDN-5D(5 A), LDN-6D(6 A), LDN-7D(7 A), LDN, and CS. LDN: Langdon; CS: Chinese Spring. The photo was acquired by Quantity One softeware (BioRad, USA).

Southern blotting is a powerful tool for copy number determination of endogenous or exogenous genes or even of low-copy-number plasmids. A southern blotting assay was used to further verify the number of homologous *ARG* genes in the wheat genome. A 525-bp DIG-labeled fragment amplified from cDNA using primers flanking a conserved *ARG* domain (Fig. 2) was used as a probe. Genomic DNA from the wheat accession CB037 was separately digested with two restriction endonucleases. After washing, three clear DNA bands were apparent in both *Bam*HI and *Kpn*I-digested genomic DNA (Fig. 4), which was in line with the number of *ARG* genes identified in the common wheat genome sequence database. To verify the chromosomal location of the *TaARG* genes, 14 disomic substitution lines were used. No amplification was observed in the LDN-2D(2 A) and LDN-2D(2B) lines when using primer pairs specific to *TaARG*-*2AS* and *TaARG-2BS*, respectively. In contrast, fragments specific to *TaARG*-*2DS* were only found in LDN-2D(2A) and LDN-2D(2B) (Fig. 5). To fine map the three *TaARG*s, the BLASTn tool was used to search mapped wheat ESTs (http://wheat.pw.usda.gov/wEST/blast/). A mapped wheat EST with an E-value = 0, BE444541, was retrieved. Based on the mapping of this EST to wheat deletion bins, the *TaARG* genes are located on chromosomes 2AS (2AS5–0.78–1.00), 2BS (2BS3-0.84-1.00), and 2DS (2DS5-0.47-1.00).

### Expression profile analysis

**Figure 6:**
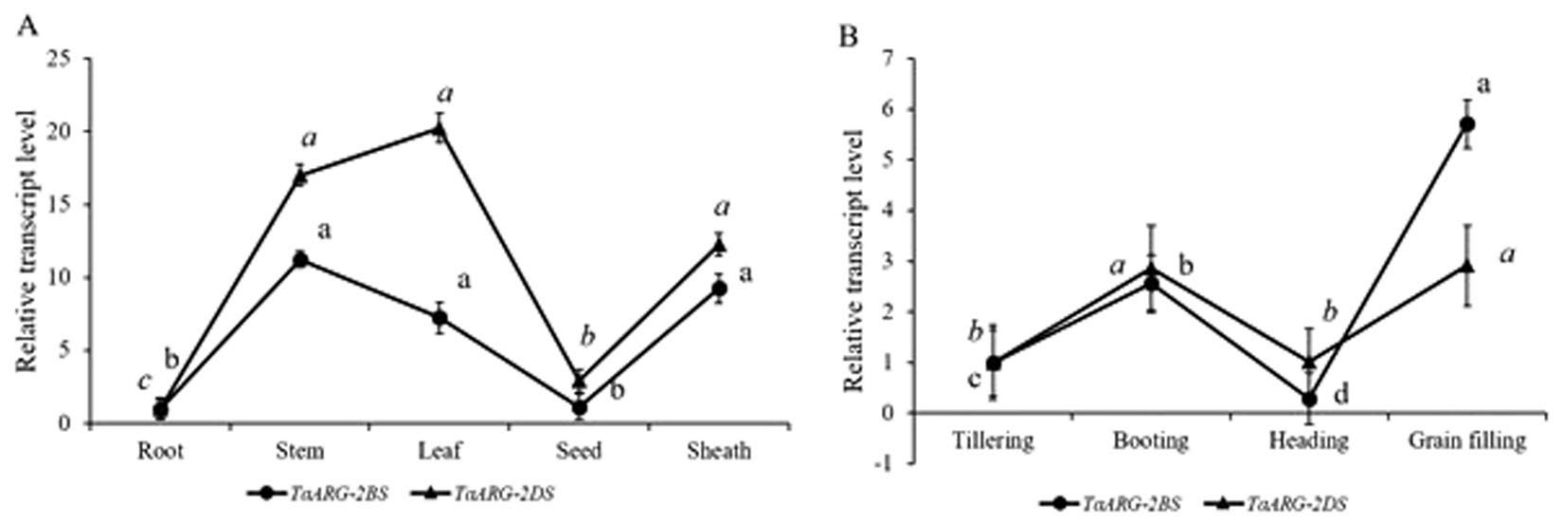
Transcript levels of two *TaARG* genes in different wheat tissues and developmental stages determined by qRT–PCR. (**A**) Different tissues; (**B**) Different developmental stages; Each data point is an average of three biological repeats (mean ± SD). Small letters in bold or italic represent significant differences between *TaARG-2BS* and *TaARG-2DS* at *α* < 0.05 using Duncan’s multiple range test.

Considering that *TaARG*-*2AS* has an in-frame stop codon and is a likely pseudogene, we did not determine its expression profile. As most plant genes show spatial and temporal expression patterns, the tissue-specific expression of the two *TaARG* genes, *TaARG*-*2BS* and *TaARG-2DS*, in the common wheat accession CB037 were determined. Both *TaARG* genes displayed the same expression trend except that the expression of *TaARG-2DS* in leaves was higher than *TaARG-2BS* (Fig. 6A). The expression level of the two *TaARG* genes in the stem, leaf, and sheath was significantly different from the level in mature seeds and roots, implying that these genes are active in aboveground tissues.

**Figure 7:**
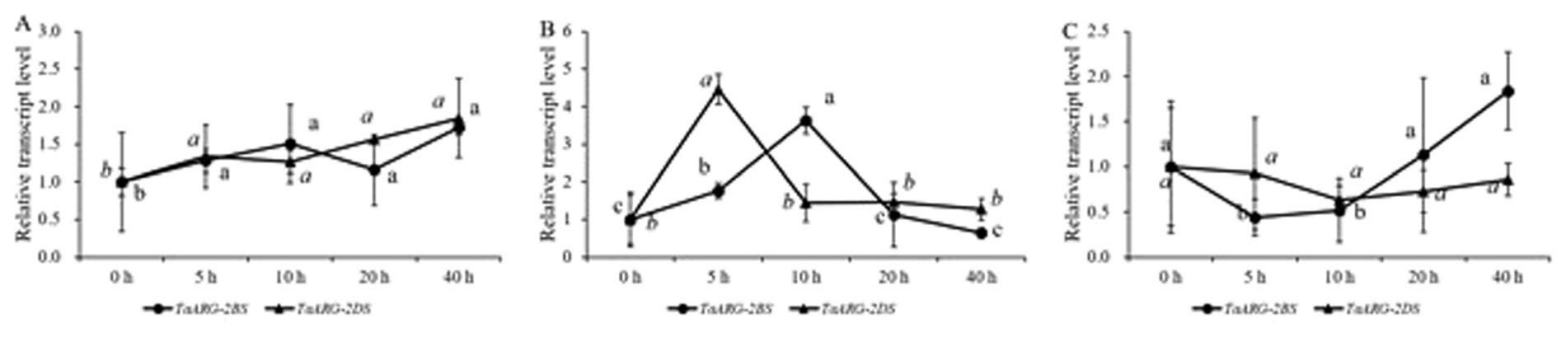
Transcript levels of *TaARG* genes in wheat leaves under abiotic stress. (**A**) Low temperature; (**B**) Drought; (**C**) NaCl. Each data point is an average of three biological repeats (mean ± SD). Small letters in bold or italic represent significant differences between *TaARG-2BS* and *TaARG-2DS* at *α* < 0.05 using Duncan’s multiple range test.

To reveal the expression pattern of wheat *TaARG* genes at different development stages, the top leaves on the main stems of CB037 at the tillering, booting, heading, and grain filling stages were collected. Transcripts of both genes quickly accumulated at the booting and grain filling stages, which is a clue to the large N requirement during these two stages (Fig. 6B). To clarify whether *TaARG* gene expression is up-regulated or down-regulated by abiotic stresses, CB037 seedlings were subjected to low temperature, drought, and NaCl treatment (Fig. 7). The expression level at 0 h was set as the reference for data analysis. For low temperature treatments, low transcript accumulation was observed for both genes, indicating that neither gene is responsive to cold treatment (Fig. 7A). In contrast, drought stress induced *TaARG*-*2DS* expression after 5 h, which was followed by continuously decreased expression. However, obvious up-regulation was not observed until 10 h for *TaARG-2BS*, which seems less sensitive to drought stress than *TaARG-2DS* (Fig. 7B). Under 100 mM NaCl stress, the expression of *TaARG-2BS* first decreased and then gradually increased over the course of stress treatment, but no significant difference in the expression of *TaARG*-*2DS* under high salinity stress was found at any time point (Fig. 7C).

**Figure 8:**
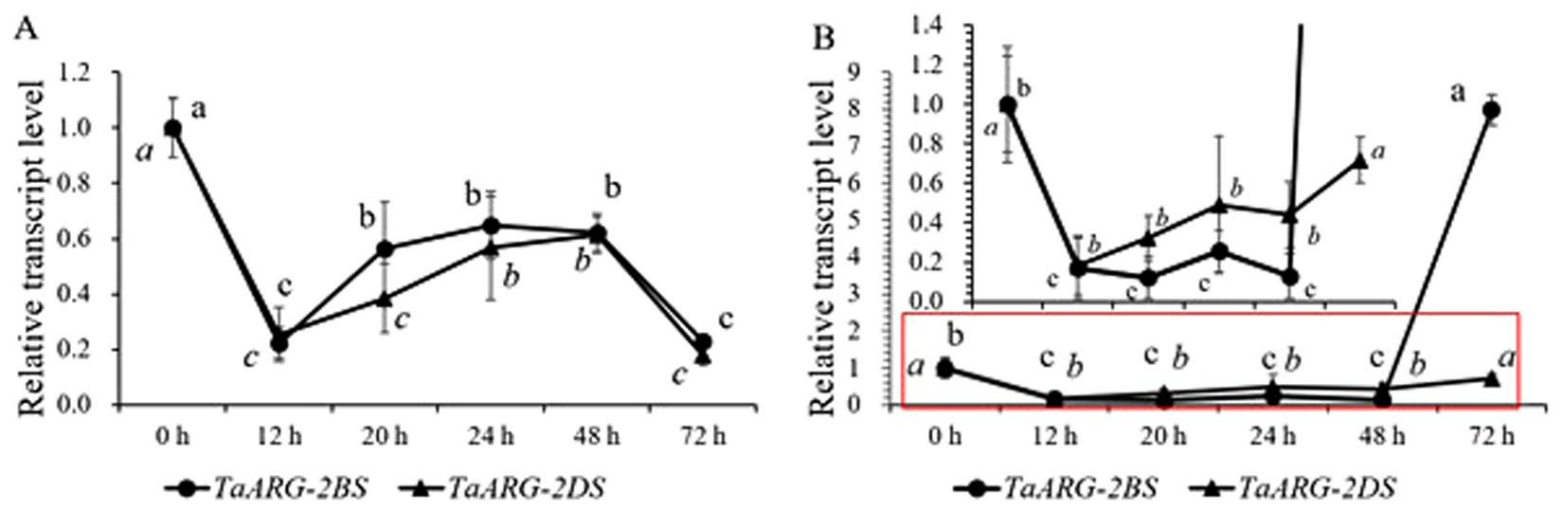
Transcript levels of *TaARG* genes in wheat leaves under inoculation of powdery mildew. (**A**) Pm97033; (**B**) Wan7107. Each data point is an average of three biological repeats (mean ± SD). Small letters in bold or italic represent significant differences between *TaARG-2BS* and *TaARG-2DS* at *α* < 0.05 using Duncan’s multiple range test. The highlighted part on the top in Fig. 8B was its partial graph in red rectangle.

The expression level of *TaARG*s under biotic stress was also assessed. The two common wheat accessions Pm97033 and Wan7107 showing opposite response to PM infection were selected and sampled at different time points after inoculation. The expression level at 0 h was set as the reference for *TaARG*s expression in the two accessions. In the PM-resistant accession Pm97033, both *TaARG-2BS* and *TaARG-2DS* displayed the same expression pattern as a mirror image of letter “N” with the highest expression at 0 h (Fig. 8A). After 12 h post inoculation (hpi), the trend of slight increase and then decrease was found for both the genes in Pm97033. However, PM induced the accumulation of *TaARG-2BS* transcripts greatly at 72 hpi in Wan7107 following a minor fluctuation at 1212–4848 hpi, which was significantly higher than that of *TaARG-2DS* transcripts at all the timepoints (Fig. 8B). During the infection span of 12 h-72 h, slow increase was found for *TaARG-2DS* (Fig. 8B). Interestingly, in the first 12 h after PM inoculation, both the genes in the two wheat accessions displayed the same downregulated expression pattern (Fig. 8).

### Biochemical and structural analysis of plant *ARG* genes

Arginase-encoding genes are wide-spread in Viridiplantae. Interestingly, all plant *ARG* genes have the same 6-exon gene structure (Additional file 2). However, copy number differs between plant groups; only one copy of *ARG* is found in diploid monocots whereas *ARG* genes in dicots form a gene cluster of 2–4 members. To determine amino acid composition, the amino acid occurrence frequency in plant ARG proteins was also determined. Polar amino acid residues make up over 38% of the total, which may be indicative of their important role in ARG catalysis. The percentage of nonpolar amino acid residues, Ala, Leu, and Val, is 27% (Additional file 3).

**Figure 9:**
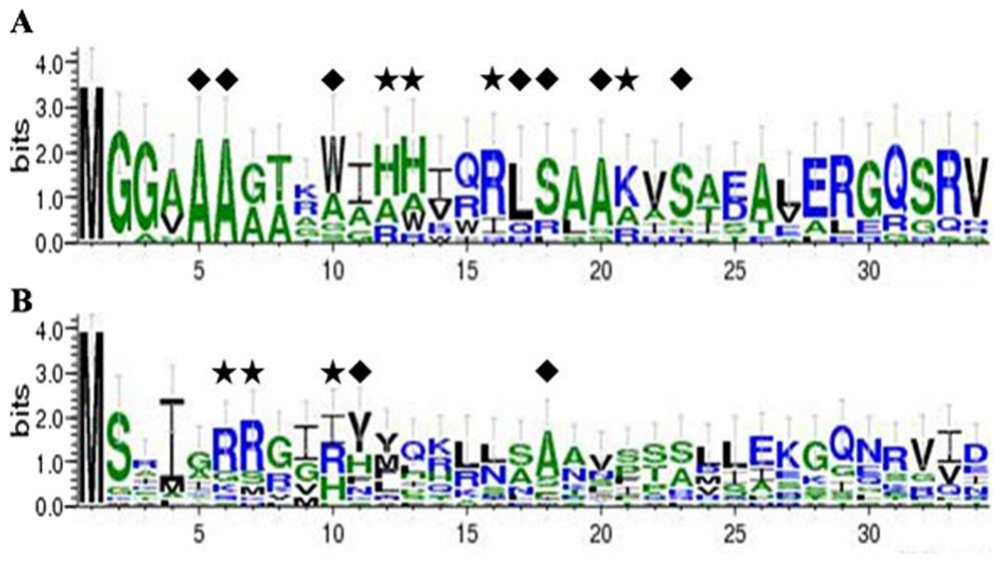
WebLogo representation of 29 mitochondrial targeting sequences. (**A**) Arginases from 11 monocotyledonous species; (**B**) Arginases from 18 dicotyledonous species: ★ indicates basic amino acid residues conserved in over 50% of sequences; ◆ indicates hydrophobic amino acid residues conserved in over 60% of sequences.

In addition, the subcellular targeting signals of a total of 43 plant ARG proteins retrieved from the Phytozome v11 database were also predicted using TargetP. Thirteen out of 43 plant ARGs didn’t have subcellular targeting signals, probably due to incomplete N-terminal sequences. The remaining plant ARGs had mitochondrial targeting signals except for one *A. lyrata* sequence (Additional file 4). Conserved motifs in the first 35 amino acid residues at the N terminus were also displayed using WebLogo software. As shown in Fig. 9, some polar amino acid residues were highly conserved in the 29 predicted ARG mitochondria-targeting sequences, although no conserved consensus motif was found. Compared to ARGs from dicotyledonous species, ARGs from monocots had more conserved amino acid residues, implying less divergence during evolution.

**Figure 10:**
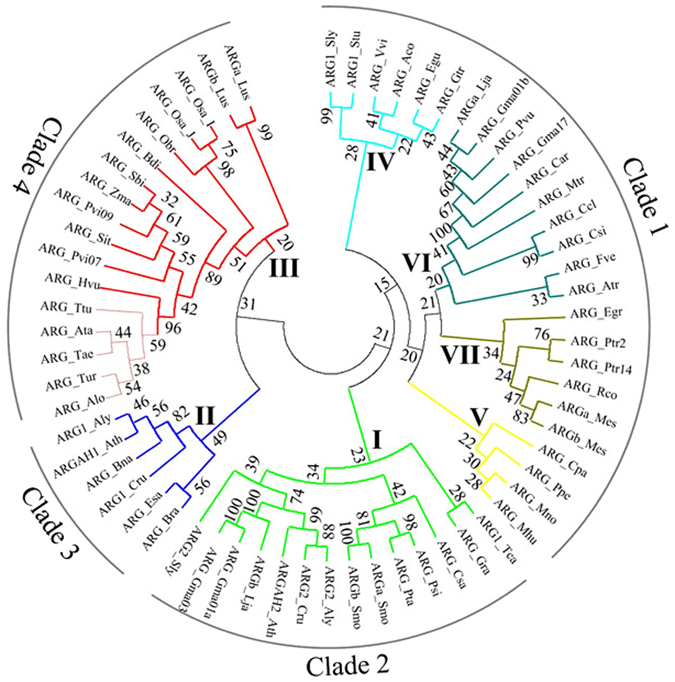
Phylogenetic tree of plant ARG proteins. The cladogram was constructed based on amino acid sequence alignments. The three letters following ARG are abbreviations for plant names in Latin (Additional file 5). The numbers at the nodes indicate the level of confidence for the major branches determined by bootstrap analysis.

To investigate the phylogenic relationships between *ARG* genes from wheat and other plant species, an unrooted phylogenetic tree was constructed from an alignment of 63 ARG amino acid sequences from diverse plant species (Additional file 5), including 15 from monocotyledonous plant species and 48 from dicotyledonous plant species. Sequences were identified by using the TaARG-2BS and TaARG-2DS amino acid sequences as queries in BLASTp searches of the non-redundant protein databases in NCBI, IWGSC, and Phytozome. To make an accurate cladogram, redundant sequences from the same plant species were removed. For example, four ARG sequences from *Theobroma cacao* were first retrieved, and three of these sequences coding for partial length proteins were excluded from the multi-sequence alignment. An initial alignment was made using ClustalW2 with default gapping penalties. After manual adjustments to the alignment, a cladogram tree was constructed and displayed using Mega 4.0. As shown in Fig. 10, there are clearly four major clades and seven phylogenetic groups (I–VII) among the plant ARG family. All the ARG proteins from monocots form a large cluster (clade 4). The ARG proteins from common wheat and its progenitor species form a small cluster (Fig. 10; indicated by light red) and share high similarity with ARG from *Hordeum vulgare*. As all plant *ARG*s share the same gene structure (Additional file 2), it may be deduced that the same gene structure is shared by their common ancestors. Compared to monocot *ARGs*, the presence of a larger group of dicot genes, forming three clades and six subfamilies, indicates that dicot *ARGs* have expanded and have more diverse functions. It is noteworthy that paralogous genes from the same species fall into different subfamilies, such as four ARGs from *Glycine max* and two each from *A. lyrata*, *A. thaliana*, *Capsella rubella*, *Lotus japonicus*, and *Solanum lycopersicum*. However, ARGs of the same plant species fall into the same cluster, such as two from *Manihot esculenta*, *Populus trichocarpa*, and *Selaginella moellendorffii*. In contrast, all the monocot ARGs, especially those from common wheat and their wild relatives, have remained highly conserved during evolution, implying that they have highly conserved functions.

## Discussion

Increasing attention has been given to N fertilizer utilization and N remobilization in wheat^23, 24^. Genetic modification has been an efficient strategy to breed wheat varieties with high N use efficiency. To date, a few genes involved in N metabolism have been cloned and functionally defined in plants, such as *NRT* ^25^, *1AMT1* ^26^ and *GS* ^27^, implying that the regulation of plant N metabolism is complex. Arg is rich in N and the key source of N during various stages of cereal growth^28^. ARG metabolizes Arg to urea, and thus participates in N remobilization following protein degradation, especially during seed germination^3, 29^. In addition, Arg catabolism also plays a positive role in several subsequent Orn-dependent pathways, including Pro-, glutamate- and Orn-dependent or -independent PA biosynthesis^30^, that modulate crop abiotic stress responses^31–33^. Considering that ARG plays an important role in N metabolism and stress response, it is important to characterize the functions of *ARG* genes.

ARG-encoding genes have been identified and characterized in a number of plant species. In this study, we successfully isolated *ARG* genes from diploid and hexaploid wheat accessions and found that only one copy of *ARG* is present in diploid genomes. Considering that the common wheat genome is composed of three heterogenous diploid genomes that merged about 8 000 years ago^34^, the presence of an *ARG* gene cluster of three copies is expected. *ARG* copy number was confirmed by southern blotting analysis. In addition, we also assigned *ARG* to specific chromosomes using Langdon-Chinese Spring substitution lines, which have been successfully applied to identify the chromosome locations of several genes, including those for grain protein content (*GPC*)^35^, fusarium head blight (*FHB*)^36^, and cadmium uptake (*Cdu1*)^37^. These lines have greater potential to identify wheat gene chromosome location compared to Chinese Spring nulli-tetrasomic lines due to the requirement of more lines for the latter. Further analysis of gene structure showed that the *ARG* gene on chromosome 2AS has a premature stop codon and belongs to a pseudogene. The presence of this stop codon was verified through resequencing of the *ARG*-2AS gene from 50 common wheat accessions. This indicates that loss of function of one copy is tolerated due to functional redundancy between *ARG* genes.

Rice and soybean ARGs have been reported to function in mitochondria^9, 38^. In this study, subcellular localization of wheat ARG proteins was predicted using TargetP, and mitochondron-targeting was further confirmed by Mitoprot (https://ihg.gsf.de/ihg/mitoprot.html) with a high probability of 0.9452. Furthermore, most plant ARGs (67%, additional file 4) are targeted to mitochondria. So, it is reasonable to believe that TaARG proteins function in mitochondria. However, there is no conserved consensus motif present in mitochondrial presequences (Fig. 9). Schneider *et al*.^39^ pointed out that the N- and C-terminal regions of different plant ARG mitochondrial presequences have a higher sequence identity than the centre regions. Analysis of conserved domains in ARG mitochondrial presequences showed that monocotyledonous ARG proteins also have this pattern of conservation (Fig. 9) while dicot ARG proteins showed less conservation, which may suggest functional diversity of ARG proteins in dicots. In addition, three conserved residues at amino acid positions 16–18 (R, L, S) that seem to be important for import into the mitochondria^4^, are more highly conserved among monocotyledonous ARGs, except for *Brachypodium distachyon*, than among dicotyledonous ARGs (Fig. 9A).

A phylogenetic tree showed that different *Arabidopsis* and tomato *ARG* genes are situated in different branches. The divergent functions of these genes have been confirmed by many researcher groups^6, 11, 12, 40^. We found high similarity between the TaARG-2BS and TaARG-2DS proteins (99.4%), implying high functional similarity between them. When aligned with OsARG, TaARG-2BS has a higher similarity to this protein than TaARG-2DS. Yang *et al*.^41^ pointed out that interspecific comparative analyses can be used to transfer known functions from rice to wheat. So, the high similarity between *TaARG-2BS* and rice *OsARG* suggests that they have overlapping functions, including N remobilization.

Analysis of gene expression profiles is of great use in dissecting the relationship between genotype and phenotype. In rice, *OsARG* is expressed at all rice growth stages in various tissues, including young and mature roots, stems, leaves, and sheaths. And *OsARG* is most highly expressed in developing panicles^9^. In this study, we also found that both *ARG* genes are expressed in developing tissues with the highest expression of *TaARG*-*2DS* in the leaf and *TaARG-2BS* in the stem and sheath. Both genes have the same low expression pattern in seeds and roots. In *Pinus taeda*, Todd *et al*.^8^ found that low levels of *ARG* transcripts accumulate in immature and mature embryos with expression level increasing following the germination of the seeds, suggesting that N remobilization is required for plant development. In contrast, *LeARG1* and *LeARG2* in tomato are most highly expressed in flower buds, but lowly expressed in full-open flowers and young fruits, and no expression is detected in healthy leaves^6^. This indicates functional differences between monocot and dicot *ARG* genes. Furthermore, the two expressed *TaARG* genes showed higher expression at the booting and grain filling stages compared to the other three developmental stages (Fig. 6B), which may partially account for large amounts of N remobilization during the booting and grain filling stages^24^.

Improvement of wheat abiotic stress tolerance has attracted a great deal of attention^42^. Thus far, many plant genes or their encoded products have been documented to be relevant to or to alleviate abiotic stresses, such as microRNAs, transcription factors (TFs), kinases, etc refs 43 and 44. ARG has been revealed as a new factor responsible for plant abiotic stress response through its effects on the levels of metabolites like PA^10, 45, 46^. In cherry tomato, some abiotic stresses such as wounding or treatment with certain hormones can stimulate *LeARG2* expression in leaves^13^. In *Arabidopsis*, *ARGAH1* and *ARGAH2* transcripts are only found in leaves, roots and cotyledons. And MeJA can induce the expression of *ARGAH2* expression but not *ARGAH1* ^40^, indicating that these paralogs have functionally diverged. Although plant *ARG*s have been shown to be induced by many abiotic stresses^6, 11–13^, whether wheat *ARG* genes have clear response to abiotic stresses have been seldom reported. As a hint, in this study, we also discovered the response of wheat *ARG* genes to abiotic stresses. That is, different *ARG* genes had different expression patterns under different abiotic stresses, especially under high salinity stress, allowing us to hypothesize that different *ARG* paralogs may respond to these stimuli differently. Furthermore, as noticed by Weinstein *et al*.^17^, wheat *ARGs* were involved in the response to some fungal disseases. To check the response of *TaARG-2BS* and *-2DS* to fungal infection, quantitative analysis on the transcripts in two wheat accessions was performed at different time duration under the infection of PM. In the PM resistance line Pm97033, the same expression pattern during all the time points after the PM infection was found for the two targeted genes compared to 0 hpi, indicating that PM infection couldn’t induce *TaARG*s expression and the PM resistance in Pm97033 is likely attributed to another gene. In contrast, *TaARG-2BS* transcripts were greatly accumulated in the PM susceptible line W7107 at 72 hpi, implying that *TaARG-2BS* is more active when PM-susceptible wheat is confronted with PM infection but the resistance seems to be submerged in the presence of a PM resistant gene in Pm97033. Pm97033 carries the *Pm21* gene from chromosome 6VS of *Dasypyrum villosum*, which was thought to confer high level of resistance to all currently known *Blumeria graminis* f. sp. *tritici* (*Bgt*) races^47^. In consideration of the different expression patterns of *TaARG*s in the resistant and susceptible wheat lines, we speculate that *TaARG*s might not be the key gene related to PM resistance in hexaploid wheat but rather actively participate in the regulatory network to alleviate the effect of PM infection, especially the *TaARG-2BS* gene. Most genes within wheat genomes are represented by two or more duplicated loci that are predominantly positioned in the proximal, low-recombination regions, and these duplicated genes constitute a complex metabolic and regulatory network^48, 49^. In the present study, we have confirmed through southern blotting analysis that there are three *TaARG* loci and found that *TaARG*-*2AS* is a pseudogene, indicating that it is not functional. Considering the complexity of the wheat genome, the functional differences among *ARG*s still need to be further investigated. In addition, *ARG* genes have recently been reported to be involved in biotic stress based on comparative interactome analyses^41, 50^. Thus, comparative genomics, detailed molecular investigations, and *in silico* modeling of a *TaARG* interaction network have great potential to reveal ARG functions and to contribute to the development of abiotic and biotic stress-tolerant wheat varieties.

## Materials and Methods

### Plant materials

In total, 52 common wheat accessions and 18 diploid progenitor accessions of common wheat were used for ARG-encoding gene isolation. In addition, a complete set of 14 Langdon–Chinese Spring chromosome substitution lines were employed to determine the chromosomal location of *ARG* genes based on Joppa and Williams^51^. The naming of Langdon–Chinese Spring substitution lines follows the format LDN-nD(nX), in which LDN is the abbreviation for the tetraploid wheat cultivar Langdon (*Triticum turgidum* L. var. *durum*), X represents the A or B chromosomes from Langdon that were replaced by D chromosomes from the hexaploid wheat Chinese Spring and n indicates the chromosome number (1–7) based on Joppa and Williams^51^. All seeds used in this study were stored at −20 °C to maintain germination potential (Additional file 1).

### Stress treatment and sample collection

All wheat seeds were surface-sterilized with 70% ethanol for 1 min, then washed 6 times with sterile water, and germinated on filter paper soaked with distilled water at 28 °C for 48 h in the dark. After germination, seedlings were transferred to pots containing sterilized vermiculite saturated with a low nutrient Hoagland’s solution (in g/L): 0.03 Ca(NO_3_)_2_·4H_2_O, 0.1 CaCl_2_·2H_2_O, 0.1 KH_2_PO_4_, 0.15 Na_2_HPO_4_·12H_2_O, 0.12 MgSO_4_·7H_2_O, 0.05 ferric citrate) and were grown under 26000 Lux for 25 d at 22 °C and a 16 h day/8 h night photoperiod with regular watering. When the primary leaves reached approximately 20 cm in length, leaves were collected for DNA and RNA extraction to isolate the *ARG* genes.

In addition, a common wheat accession CB037 was planted in the greenhouse for expression pattern analysis. In brief, the top leaves on the main stems at the tillering, booting, heading, and grain filling stages were sampled to check *ARG* gene expression levels. For detecting the expression of *ARG* genes under abiotic stresses, CB037 seedlings with primary leaves of approximately 20 cm in length were placed in a 0 °C freezer for low temperature (LT) treatment or watered using 100 mM NaCl for salt stress treatment. For drought stress treatment, the seedlings were watered with 15% PEG6000. The leaves were collected after 0, 5, 10, 20, 40 h of abiotic stress treatment and immediately frozen in liquid N for RNA extraction. Total RNA samples were also prepared from leaves, roots, stems, and leaf sheaths of CB037 plants, grown using the same plant growth protocol described above, at 5 days after heading as well as from mature seed to ascertain expression levels in different tissues.

To investigate the possible effect of PM infection on the expression of wheat *ARG* genes, two wheat accessions, Pm97033 (T6V#4 S.6DL translocation line, PM-resistant wheat accession) and Wan7107 (PM-susceptible wheat cultivar), were both subjected to inoculation of PM race E09 prevailing in Beijing area at the 3-leaf stage according to Liu *et al*.^52^. The seedlings were then covered with preservation film to maintain high humidity and placed in a growth chamber with the same photoperiod as described above and a temperature regime of 22 °C in light/20 °C in darkness. RNA was extracted from leaves sampled at 0 h, 12 h, 20 h, 24 h, 48 h, and 72 h following infection.

### Extraction of genomic DNA and total RNA, and synthesis of single-stranded cDNA

Wheat genomic DNA (gDNA) was isolated from the primary leaves (collected as described above) using CTAB as described by Gao *et al*.^53^. Gel electrophoresis was performed to check the gDNA quality and quantity using a known amount of lambda DNA as a standard. Only gDNA samples without degradation were used as templates for full-length *ARG* gene isolation and southern blotting analysis. Total RNA was extracted using the TRIzol Kit (TianGen Biotech (Beijing) Co., Ltd.) and reverse transcribed to cDNA using a PrimeScript^TM^ 1st Strand cDNA Synthesis Kit according to the manufacturer’s instructions (Dalian, China). The cDNA was stored at − 20 °C until further analysis.

### Gene sequence isolation

*OsARG* (GenBank accession number: NM001058548) was used as a query to perform tBLASTn searches of the *T. aestivum* L. EST database at the U.S. National Center for Biotechnology information (NCBI: http://www.ncbi.nlm.nih.gov) using the default parameters. The entire gene length was predicted based on annotations in the unigene database in NCBI. Sequence assembly was performed by CodonCode version 2.0.4 (CodonCode Corporation, Dedham, MA, USA; http://www.codoncode.com) and then aligned against sequences in the IWGSC database to obtain gDNA sequences. Primer pairs specific to each gene were designed according to putative 5′- and 3′-UTR (Untranslated Region) regions using DNAMAN software version 6.0 (http://www.lynnon.com) (Table 1). These primers were used to isolate the full-length *ARG* genes from common wheat and its progenitor species by polymerase chain reaction (PCR) using the extracted gDNA and cDNA described above as templates. The 20 μl PCR reaction mixture consisted of 50 ng gDNA or cDNA, 0.5 μM each primer, 0.4 mM each dNTP, 2 μl 10 × LA buffer with Mg^2+^, and 1 U LA Taq (TaKaRa, Dalian). The PCR cycling parameters were 94 °C for 4 min, followed by 35 cycles of 94 °C for 1 min, 60 °C for 1 min, 72 °C for 1–3 min, and a 10-min final extension at 72 °C in a BioRad C1000 thermal cycler (BioRad, USA). PCR products were cloned into the pEASY-T simple vector (Transgene, China). Sequencing was performed on 10 positive clones from each ligation using the ABI3700 sequencer (Applied Biosystems, CA, USA).

### Southern blotting analysis

Southern blotting analysis was performed according to Wang *et al*.^54^ to determine the copy number of *TaARG* genes. In brief, a probe was designed to hybridize to the ~250 bp upstream and downstream regions flanking the stop codon (Table 1). 10 μg CB037 gDNA was separately digested with the restriction enzymes *Bam*HI and *Kpn*I (TaKaRa, Dalian, China), which have no recognition sites in the *ARG* gDNA sequence. The 50 μl digestion reaction contained 10 μg gDNA, 5 μl 10× Buffer and was incubated overnight at 37 °C. The digested products were fractionated by 0.8% agarose gel electrophoresis and transferred onto a nylon membrane (Roche, Japan). Following incubation in prehybridization solution (5 × SSC, 0.5% sodium dodecylsulfate (SDS), 0.1% bovine serum albumin, 0.1% Ficoll, 0.1% polyvinylpyrrolidone) for 2 h at 50 °C, hybridization was performed by placing the membrane in freshly-prepared hybridization solution containing DIG-labeled probe for 20 min at 50 °C. The membrane was then washed twice at 50 °C (5 min each time) in 1 × SSC, 0.5% SDS and twice (5 min each time) at 50 °C in 0.25 × SSC, 0.5% SDS. DIG-labeled DNA probe and immunological detection were performed according to the manufacturer’s instructions (Roche DIG DNA Labeling and Detection Kit, Roche, Japan).

### Chromosome localization

Genomic DNA from the leaves of Langdon-Chinese Spring substitution lines was extracted as described above and chromosomal location was determined by AS-PCR^55^ using the gene-specific primer pairs shown in Table 1.

### Quantitative real-time RT-PCR (qRT-PCR)

Gene-specific primer pairs were designed as shown in Table 1. qRT-PCR was performed on the ABI PRISM 7300 Real-Time PCR System (ABI, Los Angeles, CA, USA) as follows: the total volume of the PCR reaction was 20 μl, containing 10 μl 2× SYBR Premix Ex Taq, 2 μl 4× diluted first-stand cDNA, 0.2 μM of each primer, 0.4 μl ROX Reference Dye II, and 6.8 μl ddH_2_O (SYBR PrimeScript RT-PCR Kit, TaKaRa, Dalian, China). The PCR samples were preheated at 95 °C for 10 min, followed by 40 cycles of amplification (95 °C for 15 s, 60 °C for 60 s). The qRT-PCR results were analyzed using the 7300 system software. A constitutively expressed wheat gene, *Actin* (GenBank Accession number: AB181991), was used for normalization of gene transcript levels. All reactions were conducted in triplicate from three biological replicates. The ΔΔC_T_ values were generated based on Schmittgen and Livak^56^. The statistical analysis of qRT-PCR experiments was performed by DPS (Data Processing System; International Business Machines Corporation: Armonk, NY, USA).

### Bioinformatics analysis

Based on the mapped ESTs in Wheat 11–2002 (nt) (http://wheat.pw.usda.gov/wEST/blast/), chromosomal location was determined by BLASTn searches against the IWGSC database. Only sequences with 100% identity to those in the IWGSC database were included in further analysis. The orthologous *ARG* genes in grass species were obtained based on sequence identity from NCBI, the Institute for Genomic Research website (TIGR: http://compbio.dfci.harvard.edu/tgi/), and Phytozome v11 (http://www.phytozome.net). Gene structures were illustrated using GSDS2.0 (Gene Structure Display Server 2.0, http://gsds.cbi.pku.edu.cn/index.php)^57^. Protein tertiary structure analysis was performed using the online SWISS-MODEL tool on the ExPASy website (https://swissmodel.expasy.org/), mitochondrial targeting peptides were predicted using TargetP (http://www.cbs.dtu.dk/services/TargetP/)^58^, and sequence conservation was illustrated using WebLogo (http://weblogo.threeplusone.com/)^59^. The BLASTp tool was employed to retrieve the homologous proteins in other plants from the NCBI database. Sequence accession numbers are listed in Additional file 2. The online ClustalW2 tool (http://www.ebi.ac.uk/Tools/clustalw2/index.html) was used for multi-sequence alignment based on the default parameters. A cladogram was constructed using Mega 4.0 software (http://www.megasoftware.net)^60^. The Neighbour-Joining method was employed with the bootstrap parameters: 1000 replicates and random seeds.

## Electronic supplementary material

Supplementary information
