# Supplementary material for: Comprehensive molecular analysis of arginase-encoding genes in common wheat and its progenitor species
Source: Sci Rep. 2017 Jul 26;7:6641. doi: 10.1038/s41598-017-07084-0 (PMC5529354; doi:10.1038/s41598-017-07084-0)
Supplement: Supplementary file 1 — Supplementary information [file 41598_2017_7084_MOESM1_ESM.docx]

**Comprehensive molecular analysis of** **arginase-encoding genes in common wheat and its progenitor species**

Maoyun She^1, 2, #^, Jing Wang^1, #^, Xinmin Wang^1, #^, Guixiang Yin^2^, Ke Wang^1^, Lipu Du^1^ & Xingguo Ye^1, *^

^1^ National Key Facility of Crop Gene Resources and Genetic Improvement/Institute of Crop Sciences, Chinese Academy of Agricultural Sciences, Beijing 100081, P. R. China. ^2^ Crop Research Institute, Anhui Academy of Agricultural Sciences, Hefei 230031, P. R. China.

^#^ These authors contributed equally to this work

^*^ Corresponding author: Xingguo Ye

Address: 12 Zhong Guan Cun South St, Haidian District, Beijing 100081, China; E-mail: yexingguo@caas.cn; Phone: 86-10-8210-5173; Fax: 86-10-8210-9765

**Supplementary material**

**Supplementary Table S1 Common wheat accessions and wheat progenitor species used in this study**

A: Hexaploid wheat accessions

| **No.** | **Wheat accession** | **Abbreviation** | **Approval/breeding year** | **No.** | **Wheat accession** | **Abbreviation** | **Approval/breeding year** |
| --- | --- | --- | --- | --- | --- | --- | --- |
| **1** | Yangmai6 | YM6 | 1984 | **27** | Ningchun47 | NC47 | 2008 |
| **2** | Xinong979 | XN979 | 1997 | **28** | Zhoumai18 | ZM18 | 2005 |
| **3** | Jimai22 | JM22 | 2006 | **29** | Neimai836 | NM836 | 2008 |
| **4** | Ningchun50 | NC50 | 2010 | **30** | Jingdong8 | JD8 | 1995 |
| **5** | Yangmai158 | YM158 | 1993 | **31** | Zhengmai9023 | ZM9023 | 2001 |
| **6** | CB037 | CB037 | — | **32** | Han6172 | H6172 | 2002 |
| **7** | Lunxuan987 | LX987 | 2003 | **33** | Shi4185 | S4185 | 2001 |
| **8** | Zhengmai366 | ZM366 | 2005 | **34** | Xiaoyan54 | XY54 | 2000 |
| **9** | Kefeng10 | KF10 | 2003 | **35** | Ningchun4 | NC4 | 1981 |
| **10** | Kefeng12 | KF12 | 2007 | **36** | Xindong20 | XD20 | 1995 |
| **11** | Chinese Spring | CS | — | **37** | Lunxuan103 | LX103 | 2010 |
| **12** | Longmai30 | LM30 | 2004 | **38** | Chuanmai42 | CM42 | 2004 |
| **13** | Verry | Verry | — | **39** | Xiaoyan216 | XY216 | 2008 |
| **14** | Zhouyuan936 | ZY936 | 2007 | **40** | Xinong9871 | XN9871 | 2008 |
| **15** | Yunong9676 | YN9676 | 2004 | **41** | Shannong138 | SN138 | 2008 |
| **16** | Yanzhan4110 | YZ4110 | 2003 | **42** | Lantian092 | LT092 | 2010 |
| **17** | Han7095 | H7095 | 2001 | **43** | Yunong211 | YN211 | 2007 |
| **18** | Hengguan35 | HG35 | 2006 | **44** | Shanmai150 | SM150 | 1999 |
| **19** | Yumai47 | YM47 | 1997 | **45** | Zhongmai895 | ZM895 | 2012 |
| **20** | Jimai21 | JM21 | 2004 | **46** | E’mai18 | EM18 | 2002 |
| **21** | Xinmai208 | XM208 | 2005 | **47** | Xinchun9 | XC9 | 1999 |
| **22** | Xinong2000 | XN2000 | 2006 | **48** | Aikang58 | AK58 | 2005 |
| **23** | Mianmai39 | MM39 | 2005 | **49** | Liangxing99 | LX99 | 2006 |
| **24** | Xiangmai55 | XM55 | 2009 | **50** | Lankao8 | LK8 | 2003 |
| **25** | Shannong19 | SN19 | 2007 | **51** | Pm97033 | Pm97033 | — |
| **26** | Zhoumai22 | ZM22 | 2007 | **52** | Wan7107 | Wan7107 | 1977 |

—: Records for government approval/breeding year were not found.

B: Diploid wheat accessions

| **No.** | **Species** | **Genome** | **Retrieval ID** |
| --- | --- | --- | --- |
| **1** | *Triticum uratu* | AA | PI 10474 |
| **2** | *Triticum monococcum* | AA^m^ | Cltr 13961 |
| **3** | *T. monococcum* | AA^m^ | Cltr 13962 |
| **4** | *T. monococcum* | AA^m^ | Cltr 13963 |
| **5** | *T. monococcum* | AA^m^ | Cltr 13964 |
| **6** | *T. monococcum* | AA^m^ | Cltr 13965 |
| **7** | *Aegilops longissima* | SS^l^ | PI 330486 |
| **8** | *A. longissima* | SS^l^ | PI 542196 |
| **9** | *A. longissima* | SS^l^ | PI 604103 |
| **10** | *A. longissima* | SS^l^ | PI 604104 |
| **11** | *Aegilops speltoides* | SS | PI 170204 |
| **12** | *A.speltoides* | SS | PI 172685 |
| **13** | *Aegilops tauschii* | DD | T26 |
| **14** | *A. tauschii* | DD | T30 |
| **15** | *A. tauschii* | DD | TD38 |
| **16** | *A. tauschii* | DD | T43 |
| **17** | *A. tauschii* | DD | T46 |
| **18** | *A. tauschii* | DD | T47 |

**Supplementary Table S2 Arginase-encoding genes from Viridiplantae**

| **Organism** | **No. of paralogous genes** | **Gene ID** | **No. of exons#** | **No. of amino acids** |
| --- | --- | --- | --- | --- |
| *Manihot esculenta* | 2 | cassava4.1_011300m | 6 | 338 |
|  |  | cassava4.1_011323m | 6 | 338 |
| *Ricinus communis* | 1 | 30174.m008853 | 6 | 338 |
| *Linum usitatissimum* | 2 | Lus10030288 | 6 | 338 |
|  |  | Lus10000795 | 6 | 338 |
| *Populus trichocarpa* | 2 | Potri.002G146200.1 | 6 | 338 |
|  |  | Potri.014G067700.1 | 6 | 333 |
| *Medicago truncatula* | 1 | Medtr4g024940.1 | 6 | 317 |
| *Phaseolus vulgaris* | 1 | Phvul.010G081600.1 | 6 | 338 |
| *Glycine max* Wm82.a2.v1* | 4 | Glyma17G131300.1 | 6 | 338 |
|  |  | Glyma17G131300.2 | 6 | 338 |
|  |  | Glyma01G140200.1 | 6 | 338 |
|  |  | Glyma03G028000.1 | 6 | 350 |
| *Cucumis sativus* | 1 | Cucsa.362680.1 | 6 | 338 |
| *Prunus persica* | 1 | ppa008288m | 6 | 338 |
| *Malus domestica* | 1 | TC77198 | 6 | 338 |
| *Fragaria vesca* | 1 | mrna03912.1-v1.0-hybrid | 6 | 338 |
| *Arabidopsis thaliana* | 2 | AT4G08870.1 | 6 | 344 |
|  |  | AT4G08900.1 | 6 | 342 |
| *Arabidopsis lyrata* | 2 | 489723 | 6 | 342 |
|  |  | 489781 | 6 | 344 |
| *Capsella rubella* | 2 | Carubv10001337m | 6 | 344 |
|  |  | Carubv10001349m | 6 | 342 |
| *Brassica rapa* FPsc v1.3* | 1 | BraraC02579 | 6 | 341 |
| *Thellungiella halophila* | 1 | Thhalv10028793m | 6 | 331 |
| *Carica papaya* | 1 | evm. model.supercontig_145.18 | 6 | 338 |
| *Gossypium raimondii* | 1 | Gorai.009G265900.1 | 6 | 349 |
| *Theobroma cacao* | 1 | Thecc1EG036209t1 | 6 | 339 |
| *Citrus sinensis* | 1 | orange1.1g019533m | 6 | 339 |
| *Citrus clementina* | 1 | Ciclev10021006m | 6 | 339 |
| *Eucalyptus grandis* | 1 | Eucgr.E01445.1 | 6 | 338 |
| *Vitis vinifera* | 1 | TC143792 | 6 | 371 |
| *Solanum lycopersicum* | 2 | Solyc01g091160.2.1 | 6 | 338 |
|  |  | Solyc01g091170.2.1 | 6 | 338 |
| *Mimulus guttatus* | 1 | mgv1a009558m | 6 | 338 |
| *Aquilegia coerulea* | 1 | Aquca_013_00658.1 | 6 | 341 |
| *Sorghum bicolor* | 1 | Sb06g000580.1 | 6 | 340 |
| *Zea mays* | 1 | GRMZM2G174671_T04 | 6 | 340 |
| *Setaria italica* | 1 | Si010532m | 6 | 340 |
| *Panicum virgatum* v1.1* | 2 | Pavir.Ib03192.1 | 6 | 419 |
|  |  | Pavir.Gb01213.1 | 6 | 378 |
| *Oryza sativa* | 1 | LOC_Os04g01590.1 | 6 | 340 |
| *Brachypodium distachyon* | 1 | Bradi5g02160.1 | 6 | 342 |
| *Selaginella moellendorffii* | 1 | 73545 | 6 | 334 |
| *Triticum monococcum* | 1 | AOTI010136064 | 6 | 340 |
| *Aegilops longissima* | nd | KJ631051 | 6 | 340 |
| *Aegilops tauschii* | 1 | AOCO010680545 | 6 | 340 |
| *Triticum turgidum* | nd | KJ631053 | 6 | 340 |
| *Triticum aestivum* | 3※ | KJ631054 | 6 | 340 |
|  |  | KJ701034 | 6 | 340 |

nd: not determined.

* Version number of database released was shown after the organism name.

# Prediction based on FGENESH+ and/or FGENESH_C online at www.softberry.com

※: Based on the results in this study

**Supplementary Table S3 The occurrence frequency of amino acids in plant arginases***

| **Plant Species** | **Gene** | **Accession No.** | **Amino acids** | | | | | | | | | | | | | | | | | | | | **Total** |
| --- | --- | --- | --- | --- | --- | --- | --- | --- | --- | --- | --- | --- | --- | --- | --- | --- | --- | --- | --- | --- | --- | --- | --- |
|  |  |  | Ala | Cys | Asp | Glu | Phe | Gly | His | Ile | Lys | Leu | Met | Asn | Pro | Gln | Arg | Ser | Thr | Val | Trp | Tyr |  |
| *Arabidopsis thaliana* | ARGAH1_Ath | NP_192629.1 | 7.02 | 1.17 | 6.43 | 7.02 | 2.92 | 9.36 | 2.34 | 6.43 | 4.97 | 9.65 | 2.34 | 3.51 | 4.68 | 2.63 | 7.31 | 8.19 | 2.63 | 9.06 | 0.29 | 2.05 | 342 |
|  | ARGAH2_Ath | NP_192626.1 | 6.69 | 0.58 | 6.40 | 7.56 | 3.20 | 9.59 | 2.62 | 5.23 | 4.94 | 10.17 | 2.91 | 2.62 | 4.94 | 2.91 | 7.27 | 6.40 | 3.78 | 9.01 | 0.58 | 2.62 | 344 |
| *Arabidopsis lyrata* | ARG1_Aly | XP_002874507.1 | 7.02 | 1.17 | 6.73 | 6.73 | 2.92 | 9.36 | 2.34 | 6.43 | 5.26 | 9.65 | 2.34 | 3.51 | 4.68 | 2.63 | 7.02 | 8.19 | 2.63 | 9.06 | 0.29 | 2.05 | 342 |
|  | ARG2_Aly | XP_002874558.1 | 6.40 | 0.58 | 6.69 | 7.27 | 3.20 | 9.30 | 2.62 | 5.23 | 4.94 | 10.17 | 2.91 | 2.91 | 4.94 | 2.62 | 7.27 | 6.98 | 3.78 | 9.01 | 0.58 | 2.62 | 344 |
| *Capsella rubella* | ARG1_Cru | XP_006288117.1 | 7.31 | 1.17 | 6.43 | 7.31 | 3.22 | 9.06 | 2.63 | 5.85 | 4.39 | 9.94 | 2.34 | 3.51 | 4.68 | 2.34 | 7.60 | 8.19 | 2.63 | 9.36 | 0.29 | 1.75 | 342 |
|  | ARG2_Cru | XP_006288104.1 | 6.40 | 0.58 | 6.40 | 7.27 | 2.62 | 9.59 | 2.62 | 5.52 | 4.65 | 9.88 | 3.20 | 3.20 | 4.36 | 3.78 | 7.27 | 6.69 | 3.49 | 9.01 | 0.58 | 2.91 | 344 |
| *Eutrema salsugineum* | ARG_Esa | XP_006397249.1 | 6.95 | 1.21 | 6.65 | 7.25 | 3.02 | 9.67 | 2.11 | 6.65 | 4.83 | 9.37 | 2.11 | 2.72 | 4.23 | 3.32 | 7.55 | 8.16 | 2.11 | 9.06 | 0.60 | 2.42 | 331 |
| *Citrus clementina* | ARG_Ccl | XP_006445281.1 | 7.67 | 0.88 | 6.78 | 6.78 | 3.24 | 9.14 | 2.65 | 5.90 | 5.01 | 9.73 | 2.36 | 3.24 | 4.72 | 2.95 | 6.78 | 6.49 | 3.54 | 10.03 | 0.29 | 1.77 | 339 |
| *Ricinus communis* | ARG_Rco | XP_002511686.1 | 7.69 | 0.89 | 6.51 | 7.10 | 3.25 | 8.88 | 2.66 | 6.21 | 5.33 | 10.06 | 2.07 | 3.25 | 4.73 | 2.96 | 7.10 | 6.80 | 3.25 | 9.17 | 0.30 | 1.78 | 338 |
| *Brassica napus* | ARG_Bna | AAK15006.1 | 6.59 | 1.20 | 6.59 | 6.59 | 2.99 | 9.58 | 2.40 | 6.59 | 4.79 | 9.58 | 2.40 | 3.59 | 4.79 | 2.69 | 7.19 | 8.08 | 2.69 | 9.28 | 0.30 | 2.10 | 334 |
| *Solanum lycopersicum* | ARG1_Sly | NP_001234578.1 | 7.40 | 0.59 | 6.21 | 7.40 | 2.96 | 9.47 | 2.96 | 5.33 | 4.73 | 9.76 | 3.25 | 3.25 | 4.73 | 3.25 | 7.10 | 7.10 | 2.96 | 9.17 | 0.30 | 2.07 | 338 |
|  | ARG2_Sly | NP_001233851.1 | 6.80 | 0.59 | 7.10 | 6.51 | 2.96 | 10.06 | 2.07 | 4.73 | 4.73 | 10.95 | 2.96 | 3.25 | 4.14 | 3.25 | 6.80 | 7.69 | 3.55 | 9.17 | 0.30 | 2.37 | 338 |
| *Solanum tuberosum* | ARG1_Stu | XP_006347881.1 | 7.40 | 0.59 | 6.51 | 7.40 | 2.66 | 9.47 | 2.66 | 5.33 | 5.03 | 9.76 | 3.25 | 3.55 | 4.73 | 3.55 | 6.51 | 6.80 | 2.96 | 9.17 | 0.30 | 2.37 | 338 |
| *Prunus persica* | ARG_Ppe | XP_007218271.1 | 7.40 | 0.89 | 6.51 | 7.10 | 3.25 | 9.76 | 2.96 | 6.80 | 3.25 | 10.36 | 2.07 | 3.55 | 4.73 | 2.66 | 7.69 | 7.10 | 2.66 | 9.17 | 0.30 | 1.78 | 338 |
| *Theobroma cacao* | ARG1_Tca | XP_007019835.1 | 7.96 | 0.88 | 6.78 | 6.49 | 2.95 | 9.44 | 2.95 | 5.01 | 4.72 | 10.91 | 2.36 | 2.95 | 4.72 | 2.95 | 7.08 | 7.96 | 2.36 | 8.85 | 0.29 | 2.36 | 339 |
| *Populus trichocarpa* | ARG_Ptr02 | XP_002301277.2 | 6.51 | 1.18 | 6.51 | 7.10 | 2.96 | 9.17 | 2.66 | 6.21 | 5.33 | 10.65 | 2.07 | 3.25 | 5.03 | 3.25 | 7.10 | 6.51 | 3.55 | 8.88 | 0.30 | 1.78 | 338 |
|  | ARG_Ptr14 | XP_002320051.1 | 7.21 | 1.20 | 6.61 | 7.21 | 3.00 | 9.31 | 3.00 | 5.71 | 5.11 | 10.81 | 2.40 | 3.30 | 5.11 | 3.00 | 6.61 | 6.61 | 2.70 | 9.01 | 0.30 | 1.80 | 333 |
| *Fragaria vesca* | ARG_Fve | XP_004306935.1 | 6.80 | 0.89 | 6.80 | 6.51 | 2.96 | 9.17 | 3.25 | 5.33 | 4.73 | 10.06 | 2.37 | 3.55 | 5.03 | 2.66 | 7.40 | 7.40 | 2.66 | 10.06 | 0.30 | 2.07 | 338 |
| *Vitis vinifera* | ARG_Vvi | CBI38896.3 | 7.55 | 0.81 | 5.93 | 6.74 | 3.50 | 8.89 | 3.50 | 6.20 | 4.31 | 9.70 | 2.70 | 4.58 | 4.58 | 2.96 | 7.01 | 7.28 | 3.23 | 8.36 | 0.54 | 1.62 | 371 |
| *Morus notabilis* | ARG_Mno | EXB76234.1 | 7.65 | 0.59 | 6.18 | 6.76 | 3.53 | 9.41 | 2.94 | 5.88 | 5.59 | 9.71 | 2.35 | 4.12 | 4.71 | 2.94 | 6.47 | 6.76 | 3.24 | 9.12 | 0.29 | 1.76 | 340 |
| *Malus hupehensis* | ARG_Mhu | ABR13881.1 | 7.40 | 0.89 | 6.80 | 6.21 | 3.55 | 9.17 | 3.25 | 5.92 | 4.14 | 10.06 | 2.07 | 3.85 | 4.73 | 2.96 | 7.40 | 6.80 | 2.66 | 10.06 | 0.30 | 1.78 | 338 |
| *Eucalyptus grandis* | ARG_Egr | KCW73011.1 | 7.69 | 0.89 | 7.10 | 7.40 | 2.96 | 8.88 | 2.66 | 5.33 | 4.14 | 10.36 | 2.37 | 3.25 | 4.44 | 2.66 | 8.28 | 6.51 | 3.25 | 9.76 | 0.30 | 1.78 | 338 |
| *Sorghum bicolor* | ARG_Sbi | XP_002446022.1 | 8.24 | 1.18 | 6.18 | 7.94 | 2.65 | 9.41 | 2.65 | 5.00 | 5.00 | 9.71 | 2.06 | 2.35 | 4.41 | 2.94 | 7.06 | 7.35 | 3.82 | 9.71 | 0.59 | 1.76 | 340 |
| *Oryza brachyantha* | ARG_Obr | XP_006652085.1 | 8.53 | 1.18 | 6.76 | 7.35 | 2.65 | 9.41 | 2.65 | 5.29 | 4.71 | 9.71 | 2.06 | 2.35 | 4.41 | 2.35 | 7.65 | 7.35 | 3.24 | 10.00 | 0.59 | 1.76 | 340 |
| *Oryza sativa Indica Group* | ARG_Osa_I | B8AU84.1 | 7.94 | 1.18 | 6.76 | 7.35 | 2.65 | 9.71 | 2.65 | 5.29 | 4.71 | 9.71 | 2.06 | 2.35 | 4.41 | 2.35 | 7.65 | 7.35 | 3.53 | 10.00 | 0.59 | 1.76 | 340 |
| *Oryza sativa Japonica Group* | ARG_Osa_J | NP_001052013.1 | 8.24 | 1.18 | 6.76 | 7.35 | 2.65 | 9.71 | 2.65 | 5.29 | 4.71 | 9.71 | 2.06 | 2.35 | 4.41 | 2.35 | 7.65 | 7.35 | 3.24 | 10.00 | 0.59 | 1.76 | 340 |
| *Erythranthe guttata* | ARG_Egu | EYU31976.1 | 6.21 | 0.89 | 6.51 | 6.80 | 2.96 | 9.76 | 2.96 | 5.92 | 5.03 | 10.65 | 2.66 | 3.85 | 4.73 | 2.66 | 7.40 | 6.80 | 3.25 | 9.17 | 0.30 | 1.48 | 338 |
| *Setaria italica* | ARG_Sit | XP_004974987.1 | 8.53 | 1.18 | 6.47 | 7.65 | 2.65 | 9.41 | 2.65 | 5.00 | 5.00 | 9.71 | 2.06 | 2.65 | 4.41 | 2.94 | 7.06 | 7.35 | 3.24 | 9.71 | 0.59 | 1.76 | 340 |
| *Gentiana triflora* | ARG_Gtr | BAI22841.1 | 6.80 | 0.89 | 5.92 | 7.40 | 3.25 | 9.47 | 2.96 | 5.92 | 4.14 | 10.06 | 2.66 | 4.44 | 5.33 | 3.25 | 7.10 | 7.10 | 2.37 | 8.88 | 0.30 | 1.78 | 338 |
| *Cucumis sativus* | ARG_Csa | XP_004145005.1 | 7.40 | 0.89 | 6.51 | 7.40 | 2.96 | 9.47 | 2.07 | 5.33 | 4.44 | 9.76 | 2.37 | 3.55 | 4.73 | 2.96 | 7.40 | 7.10 | 2.66 | 10.06 | 0.30 | 2.66 | 338 |
| *Zea mays* | ARG_Zma | ACF85494.1 | 8.53 | 1.18 | 5.88 | 7.94 | 2.65 | 9.41 | 2.94 | 5.00 | 5.00 | 9.71 | 2.06 | 2.65 | 4.41 | 2.65 | 7.06 | 7.35 | 3.53 | 9.71 | 0.59 | 1.76 | 340 |
| *Cicer arietinum* | ARG_Car | XP_004506527.1 | 8.88 | 0.89 | 6.80 | 6.21 | 3.25 | 9.17 | 3.25 | 5.62 | 3.85 | 10.06 | 2.07 | 3.25 | 4.14 | 2.96 | 7.40 | 6.51 | 3.55 | 10.06 | 0.30 | 1.78 | 338 |
| *Hordeum vulgare* | ARG_Hvu | BAJ96580.1 | 9.06 | 1.17 | 7.02 | 7.02 | 2.63 | 9.94 | 2.05 | 4.97 | 4.09 | 9.65 | 2.34 | 2.63 | 4.39 | 2.92 | 7.89 | 7.31 | 3.51 | 9.06 | 0.58 | 1.75 | 342 |
| *Amborella trichopoda* | ARG_Atr | XP_006838611.1 | 7.12 | 1.19 | 7.42 | 5.93 | 3.56 | 9.20 | 2.67 | 4.75 | 5.04 | 10.98 | 2.67 | 3.26 | 4.75 | 3.26 | 6.53 | 6.53 | 4.15 | 9.20 | 0.30 | 1.48 | 337 |
| *Picea sitchensis* | ARG_Psi | ABK23295.1 | 7.04 | 1.17 | 5.87 | 7.33 | 3.52 | 8.80 | 2.93 | 5.57 | 5.57 | 10.26 | 3.52 | 2.64 | 4.69 | 3.52 | 6.74 | 7.04 | 3.81 | 8.21 | 0.29 | 1.47 | 341 |
| *Lotus japonicus* | ARGa_Lja | AFK40675.1 | 7.69 | 0.89 | 7.10 | 6.51 | 3.25 | 9.17 | 3.55 | 6.21 | 4.14 | 9.17 | 2.96 | 3.55 | 4.44 | 2.37 | 7.10 | 6.51 | 3.55 | 9.76 | 0.30 | 1.78 | 338 |
|  | ARGb_Lja | AK339774.1 | 8.45 | 0.87 | 6.12 | 7.29 | 3.21 | 9.91 | 3.21 | 5.25 | 4.66 | 9.04 | 3.21 | 2.92 | 4.66 | 3.50 | 6.12 | 7.00 | 2.62 | 8.16 | 0.29 | 3.50 | 343 |
| *Phaseolus vulgaris* | ARG_Pvu | XP_007134852.1 | 8.58 | 0.89 | 7.10 | 6.51 | 3.25 | 9.47 | 2.66 | 6.21 | 4.44 | 9.76 | 2.66 | 2.96 | 4.44 | 2.37 | 7.10 | 6.80 | 3.55 | 9.47 | 0.30 | 1.48 | 338 |
| *Brachypodium distachyon* | ARG_Bdi | XP_003580734.1 | 9.65 | 1.17 | 7.31 | 7.31 | 2.63 | 9.65 | 2.34 | 5.26 | 3.80 | 9.65 | 2.63 | 2.05 | 4.68 | 2.92 | 7.60 | 7.31 | 3.22 | 8.77 | 0.58 | 1.46 | 342 |
| *Pinus taeda* | ARG_Pta | AAK07744.1 | 7.33 | 1.17 | 6.16 | 6.74 | 3.23 | 9.38 | 2.93 | 4.99 | 5.87 | 9.97 | 4.11 | 2.35 | 4.69 | 3.52 | 6.16 | 7.62 | 3.52 | 8.50 | 0.29 | 1.47 | 341 |
| *Medicago truncatula* | ARG_Mtr | XP_003605167.1 | 7.90 | 0.91 | 7.29 | 6.38 | 3.34 | 8.81 | 3.34 | 4.86 | 3.95 | 10.33 | 2.43 | 4.26 | 4.26 | 3.04 | 6.99 | 6.69 | 3.04 | 9.73 | 0.30 | 2.13 | 329 |
| *Selaginella moellendorffii* | ARGa_Smo | XP_002960232.1 | 8.38 | 1.20 | 6.29 | 6.29 | 2.69 | 9.28 | 3.29 | 5.69 | 5.09 | 8.98 | 2.99 | 2.99 | 4.19 | 4.19 | 5.99 | 7.49 | 3.89 | 8.98 | 0.60 | 1.50 | 334 |
|  | ARGb_Smo | XP_002967483.1 | 8.68 | 1.20 | 6.29 | 6.29 | 2.69 | 9.28 | 3.29 | 5.69 | 5.09 | 8.98 | 2.99 | 2.99 | 4.19 | 4.19 | 5.99 | 7.49 | 3.89 | 8.68 | 0.60 | 1.50 | 334 |
| *Citrus sinensis* | ARG_Csi | KDO85792.1 | 7.25 | 0.48 | 7.25 | 7.25 | 4.35 | 8.70 | 3.38 | 5.31 | 4.83 | 9.18 | 2.90 | 2.90 | 4.35 | 2.90 | 6.28 | 6.28 | 2.90 | 11.11 | 0.00 | 2.42 | 207 |
| *Glycine max* | ARG_Gma01a | ACU21474.1 | 6.57 | 1.43 | 6.86 | 7.14 | 4.00 | 9.43 | 2.57 | 5.43 | 6.00 | 9.14 | 3.71 | 3.43 | 4.29 | 2.29 | 6.86 | 7.71 | 2.29 | 8.00 | 0.29 | 2.57 | 350 |
|  | ARG_Gma01b | XP_003517040.1 | 8.58 | 1.18 | 7.10 | 6.51 | 2.96 | 9.17 | 2.96 | 6.21 | 4.44 | 10.06 | 2.37 | 2.96 | 4.44 | 2.66 | 7.40 | 6.51 | 3.25 | 9.17 | 0.30 | 1.78 | 338 |
|  | ARG_Gma03 | NP_001237121.1 | 6.57 | 1.71 | 6.86 | 7.14 | 4.00 | 9.71 | 2.29 | 5.43 | 5.71 | 9.14 | 3.71 | 3.71 | 4.86 | 2.29 | 6.86 | 7.43 | 2.00 | 7.71 | 0.29 | 2.57 | 350 |
|  | ARG_Gma17 | XP_003549862.1 | 8.88 | 1.18 | 6.80 | 6.51 | 3.25 | 9.17 | 2.37 | 5.92 | 4.44 | 10.06 | 2.37 | 2.96 | 4.73 | 2.66 | 7.40 | 6.21 | 3.55 | 9.47 | 0.30 | 1.78 | 338 |
| *Panicum virgatum* | ARG_Pvi07 | Pavir.Gb01213.1 | 7.51 | 1.69 | 5.81 | 7.02 | 3.63 | 10.17 | 2.42 | 4.84 | 4.84 | 9.93 | 2.18 | 2.18 | 4.84 | 2.66 | 6.78 | 7.75 | 3.87 | 9.44 | 0.48 | 1.94 | 413 |
|  | ARG_Pvi09 | Pavir.Ib03192.1 | 8.24 | 1.18 | 6.47 | 7.65 | 2.65 | 9.41 | 2.65 | 5.00 | 5.00 | 9.71 | 2.06 | 2.35 | 4.41 | 2.94 | 7.06 | 7.35 | 3.82 | 9.71 | 0.59 | 1.76 | 340 |
| *Aquilegia coerulea* | ARG_Aco | Aquca_013_00658.1 | 7.62 | 1.17 | 7.62 | 5.28 | 2.93 | 9.38 | 3.23 | 6.16 | 5.28 | 9.97 | 2.05 | 3.23 | 4.69 | 2.35 | 6.74 | 7.33 | 3.52 | 9.09 | 0.59 | 1.76 | 341 |
| *Carica papaya* | ARG_Cpa | contig_145.18 | 7.69 | 0.89 | 6.51 | 6.51 | 2.96 | 9.76 | 2.66 | 5.92 | 4.14 | 10.06 | 2.07 | 3.55 | 4.73 | 3.25 | 7.10 | 6.80 | 3.55 | 9.76 | 0.30 | 1.78 | 338 |
| *Manihot esculenta* | ARGa_Mes | cassava4.1_011300m | 7.40 | 0.89 | 6.51 | 6.80 | 3.25 | 9.17 | 2.66 | 6.51 | 4.73 | 10.36 | 2.07 | 3.25 | 4.73 | 3.25 | 7.40 | 6.51 | 3.25 | 9.47 | 0.30 | 1.48 | 338 |
|  | ARGb_Mes | cassava4.1_011323m | 8.28 | 0.89 | 6.51 | 6.80 | 3.25 | 9.17 | 2.66 | 6.21 | 4.14 | 10.36 | 2.07 | 3.55 | 4.73 | 3.25 | 7.10 | 6.80 | 2.96 | 9.76 | 0.30 | 1.18 | 338 |
| *Brassica rapa* | ARG_Bra | Bra000659 | 6.45 | 1.17 | 6.45 | 7.33 | 2.93 | 9.68 | 2.35 | 5.57 | 4.99 | 9.97 | 2.05 | 2.64 | 4.40 | 2.93 | 7.62 | 8.50 | 3.23 | 9.68 | 0.29 | 1.76 | 341 |
| *Linum usitatissimum* | ARGa_Lus | Lus10030288 | 7.40 | 1.18 | 7.10 | 7.40 | 2.96 | 8.58 | 2.66 | 5.92 | 4.14 | 10.36 | 2.37 | 3.25 | 4.73 | 2.66 | 7.69 | 5.92 | 3.85 | 9.76 | 0.30 | 1.78 | 338 |
|  | ARGb_Lus | Lus10000795 | 7.40 | 1.18 | 7.10 | 7.40 | 2.96 | 8.58 | 2.66 | 5.62 | 4.73 | 10.65 | 2.37 | 3.25 | 4.73 | 2.37 | 7.40 | 5.92 | 3.85 | 9.76 | 0.30 | 1.78 | 338 |
| *Gossypium raimondii* | ARG_Gra | Gorai.009G265900.1 | 7.45 | 1.43 | 6.88 | 6.88 | 4.30 | 9.17 | 2.58 | 5.16 | 4.58 | 10.32 | 2.58 | 3.15 | 4.58 | 2.29 | 6.59 | 7.45 | 3.44 | 9.17 | 0.29 | 1.72 | 349 |
| *Triticum aestivum* | ARG_Tae | AGR45902.1 | 8.53 | 1.18 | 6.76 | 7.35 | 2.65 | 10.00 | 2.06 | 5.00 | 4.12 | 9.71 | 2.35 | 2.35 | 4.41 | 2.94 | 7.94 | 7.35 | 3.82 | 9.12 | 0.59 | 1.76 | 340 |
| *Triticum turgidum* | ARG_Ttu | KJ631053 | 8.53 | 1.18 | 6.76 | 7.35 | 2.65 | 10.00 | 2.06 | 5.00 | 4.12 | 9.71 | 2.35 | 2.35 | 4.41 | 2.94 | 7.94 | 7.35 | 3.82 | 9.12 | 0.59 | 1.76 | 340 |
| *Triticum urartu* | ARG_Tur | EMS66394.1 | 8.50 | 1.17 | 6.74 | 7.33 | 2.64 | 10.26 | 2.05 | 4.99 | 4.11 | 9.68 | 2.35 | 2.35 | 4.40 | 2.93 | 7.92 | 7.33 | 3.81 | 9.09 | 0.59 | 1.76 | 341 |
| *Aegilops longissima* | ARG_Alo | KJ631051 | 8.53 | 1.18 | 6.76 | 7.35 | 2.65 | 10.00 | 2.06 | 5.29 | 4.12 | 9.71 | 2.06 | 2.35 | 4.41 | 2.94 | 7.94 | 7.35 | 3.82 | 9.12 | 0.59 | 1.76 | 340 |
| *Aegilops tauschii* | ARG_Ata | EMT05347.1 | 10.94 | 1.02 | 6.11 | 7.12 | 2.29 | 9.67 | 1.78 | 5.34 | 4.07 | 9.92 | 2.04 | 2.04 | 4.33 | 3.05 | 8.65 | 7.38 | 3.56 | 8.40 | 0.76 | 1.53 | 393 |
| **Average** |  |  | 7.71 | 1.04 | 6.63 | 7.00 | 3.06 | 9.42 | 2.69 | 5.58 | 4.69 | 9.92 | 2.50 | 3.09 | 4.60 | 2.91 | 7.18 | 7.14 | 3.27 | 9.26 | 0.40 | 1.90 | 339.83 |

*The frequencies are given in percent.

**Supplementary Table S4 Prediction of mitochondrial targeting using TargetP**

| **Plant species** | **Length (aa)** | **cTP** | **mTP** | **SP** | **other** | **Localization*** |
| --- | --- | --- | --- | --- | --- | --- |
| *Aegilops tauschii* | 393 | 0.291 | 0.659 | 0.005 | 0.048 | M |
| *Amborella trichopoda* | 337 | 0.11 | 0.115 | 0.018 | 0.666 | _ |
| *Arabidopsis lyrata* | 342 | 0.282 | 0.239 | 0.007 | 0.181 | C |
| *Arabidopsis thaliana 1* | 342 | 0.213 | 0.47 | 0.004 | 0.117 | M |
| *Arabidopsis thaliana 2* | 344 | 0.027 | 0.833 | 0.01 | 0.045 | M |
| *Brachypodium distachyon* | 342 | 0.344 | 0.365 | 0.019 | 0.072 | M |
| *Brassica napus* | 334 | 0.215 | 0.467 | 0.004 | 0.116 | M |
| *Capsella rubella* | 342 | 0.139 | 0.421 | 0.01 | 0.081 | M |
| *Cicer arietinum* | 338 | 0.09 | 0.665 | 0.01 | 0.088 | M |
| *Citrus clementina* | 339 | 0.051 | 0.3 | 0.026 | 0.428 | _ |
| *Cucumis sativus* | 338 | 0.118 | 0.467 | 0.01 | 0.143 | M |
| *Eutrema salsugineum* | 331 | 0.079 | 0.394 | 0.01 | 0.209 | M |
| *Fragaria vesca* | 338 | 0.092 | 0.601 | 0.004 | 0.247 | M |
| *Gentiana triflora* | 338 | 0.115 | 0.146 | 0.024 | 0.496 | _ |
| *Glycine max* | 338 | 0.076 | 0.577 | 0.01 | 0.23 | M |
| *Hordeum vulgare* | 342 | 0.41 | 0.638 | 0.008 | 0.02 | M |
| *Lotus japonicus* | 338 | 0.186 | 0.321 | 0.02 | 0.243 | M |
| *Malus hupehensis* | 338 | 0.075 | 0.207 | 0.018 | 0.463 | _ |
| *Medicago truncatula* | 338 | 0.224 | 0.485 | 0.012 | 0.085 | M |
| *Mimulus guttatus* | 338 | 0.192 | 0.111 | 0.014 | 0.709 | _ |
| *Morus notabilis* | 340 | 0.119 | 0.09 | 0.032 | 0.64 | _ |
| *Oryza brachyantha* | 340 | 0.128 | 0.811 | 0.004 | 0.105 | M |
| *Oryza sativa* Indica Group | 340 | 0.128 | 0.811 | 0.004 | 0.105 | M |
| *Oryza sativa* Japonica Group | 340 | 0.128 | 0.811 | 0.004 | 0.105 | M |
| *Phaseolus vulgaris* | 338 | 0.146 | 0.318 | 0.027 | 0.174 | M |
| *Picea sitchensis* | 341 | 0.073 | 0.553 | 0.012 | 0.1 | M |
| *Pinus taeda* | 341 | 0.087 | 0.632 | 0.008 | 0.089 | M |
| *Populus trichocarpa* | 338 | 0.099 | 0.138 | 0.022 | 0.645 | _ |
| *Prunus persica* | 338 | 0.124 | 0.162 | 0.021 | 0.453 | _ |
| *Ricinus communis* | 338 | 0.041 | 0.332 | 0.018 | 0.37 | _ |
| *Selaginella moellendorffii* | 334 | 0.108 | 0.255 | 0.027 | 0.23 | M |
| *Setaria italica* | 340 | 0.276 | 0.496 | 0.01 | 0.156 | M |
| *Solanum lycopersicum 1* | 338 | 0.147 | 0.322 | 0.012 | 0.453 | _ |
| *Solanum lycopersicum 2* | 338 | 0.276 | 0.074 | 0.03 | 0.623 | _ |
| *Solanum tuberosum* | 338 | 0.15 | 0.206 | 0.015 | 0.586 | _ |
| *Sorghum bicolor* | 340 | 0.317 | 0.46 | 0.008 | 0.173 | M |
| *Theobroma cacao 1* | 339 | 0.127 | 0.354 | 0.017 | 0.311 | M |
| *Theobroma cacao 3* | 237 | 0.127 | 0.354 | 0.017 | 0.311 | M |
| *Theobroma cacao 4* | 288 | 0.127 | 0.354 | 0.017 | 0.311 | M |
| *Triticum aestivum* | 340 | 0.386 | 0.739 | 0.006 | 0.03 | M |
| *Triticum urartu* | 340 | 0.287 | 0.784 | 0.004 | 0.047 | M |
| *Vitis vinifera* | 371 | 0.181 | 0.138 | 0.019 | 0.755 | _ |
| *Zea mays* | 340 | 0.271 | 0.498 | 0.009 | 0.157 | M |

cTP: a chloroplast transit peptide;

mTP: a mitochondrial targeting peptide;

SP: a signal peptide;

*: C, M, S, and _ mean cTP, mTP, SP, and any other location, respectively.

The names in red mean those without predicted mitochondrial targeting signals.

**Supplementary Table S5 Accession numbers of ARG protein sequences used in phylogenetic analysis**

| **Plant species** | **Accession Nos** | **No of amino acid residue** | **Annotation*** |
| --- | --- | --- | --- |
| *Aegilops longissima* | KJ631051 | 340 aa | ARG_Alo |
| *Aegilops tauschii* | EMT05347.1 | 393 aa | ARG_Ata |
| *Amborella trichopoda* | XP_006838611.1 | 337 aa | ARG_Atr |
| *Aquilegia coerulea* | Aquca_013_00658.1 | 341 aa | ARG-Aco |
| *Arabidopsis lyrata ssp. lyrata* | XP_002874507.1 | 342 aa | ARG1_Aly |
| *Arabidopsis lyrata subsp. lyrata* | XP_002874558.1 | 344 aa | ARG2_Aly |
| *Arabidopsis thaliana* | NP_192629.1 | 342 aa | arginine amidohydrolase 1; ARGAH1_Ath |
| *Arabidopsis thaliana* | NP_192626.1 | 344 aa | arginine amidohydrolase 2; ARGAH2_Ath |
| *Brachypodium distachyon* | XP_003580734.1 | 342 aa | ARG_Bdi |
| *Brassica napus* | AAK15006.1 | 334 aa | ARG_Bna |
| *Brassica rapa* | XP_009134113.1 | 341 aa | ARG_Bra |
| *Capsella rubella* | XP_006288117.1 | 342 aa | ARG1_Cru |
| *Capsella rubella* | XP_006288104.1 | 344 aa | ARG2_Cru |
| *Carica papaya* | contig_145.18 | 338 aa | ARG_Cpa |
| *Cicer arietinum* | XP_004506527.1 | 338 aa | ARG_Car |
| *Citrus clementina* | XP_006445281.1 | 339 aa | ARG_Ccl |
| *Citrus sinensis* | KDO85792.1 | 207 aa | ARG_Csi |
| *Cucumis sativus* | XP_004145005.1 | 338 aa | ARG_Csa |
| *Erythranthe guttata* | EYU31976.1 | 338 aa | ARG_Egu |
| *Eucalyptus grandis* | KCW73011.1 | 338 aa | ARG_Egr |
| *Eutrema salsugineum* | XP_006397249.1 | 331 aa | ARG_Esa |
| *Fragaria vesca ssp. vesca* | XP_004306935.1 | 338 aa | ARG_Fve |
| *Gentiana triflora* | BAI22841.1 | 338 aa | ARG_Gtr |
| *Glycine max* | ACU21474.1 | 350 aa | chr01g33635; ARG_Gma01a |
| *Glycine max* | XP_003517040.1 | 338 aa | chr01g33761; ARG_Gma01b |
| *Glycine max* | NP_001237121.1 | 350 aa | chr03g03270; ARG_Gma03 |
| *Glycine max* | XP_003549862.1 | 338 aa | chr17g14040; ARG_Gma17 |
| *Gossypium raimondii* | KJB59654.1 | 349 aa | ARG_Gra |
| *Hordeum vulgare ssp. vulgare* | BAJ96580.1 | 342 aa | ARG_Hvu |
| *Linum usitatissimum* | Lus10030288 | 338 aa | ARGa_Lus |
| *Linum usitatissimum* | Lus10000795 | 338 aa | ARGb_Lus |
| *Lotus japonicus* | AFK40675.1 | 338 aa | ARGa_Lja |
| *Lotus japonicus* | AK339774.1 | 343 aa | ARGb_Lja |
| *Malus hupehensis* | ABR13881.1 | 338 aa | ARG_Mhu |
| *Manihot esculenta* | cassava4.1_011300m | 338 aa | ARGa_Mes |
| *Manihot esculenta* | OAY49327.1 | 338 aa | MANES_05G047100; ARGb_Mes |
| *Medicago truncatula* | AFK41313.1 | 338 aa | ARG_Mtr |
| *Morus notabilis* | EXB76234.1 | 340 aa | ARG_Mno |
| *Oryza brachyantha* | XP_006652085.1 | 340 aa | arginase 1; ARG_Obr |
| *Oryza sativa Indica Group* | ADK74000.1 | 340 aa | ARG_Osa_I |
| *Oryza sativa Japonica Group* | NP_001052013.1 | 340 aa | ARG_Osa_J |
| *Panicum virgatum* | Pavir.Gb01213.1 | 417 aa | ARG_Pvi07 |
| *Panicum virgatum* | Pavir.Ib03192.1 | 340 aa | ARG_Pvi09 |
| *Phaseolus vulgaris* | XP_007134852.1 | 338 aa | ARG_Pvu |
| *Picea sitchensis* | ABK26575.1 | 341 aa | ARG_Psi |
| *Pinus taeda* | AAK07744.1 | 341 aa | ARG_Pta |
| *Populus trichocarpa* | XP_002301277.2 | 338 aa | ARG_Ptr2 |
| *Populus trichocarpa* | XP_002320051.1 | 333 aa | ARG_Ptr14 |
| *Prunus persica* | XP_007218271.1 | 338 aa | ARG_Ppe |
| *Ricinus communis* | XP_002511686.1 | 338 aa | ARG_Rco |
| *Selaginella moellendorffii* | XP_002960232.1 | 334 aa | ARGa_Smo |
| *Selaginella moellendorffii* | XP_002967483.1 | 334 aa | ARGb_Smo |
| *Setaria italica* | XP_004974987.1 | 340 aa | ARG_Sit |
| *Solanum lycopersicum* | NP_001234578.1 | 338 aa | arginase 1; ARG1_Sly |
| *Solanum lycopersicum* | NP_001233851.1 | 338 aa | arginase 2; ARG2_Sly |
| *Solanum tuberosum* | XP_006347881.1 | 338 aa | arginase 1; ARG1_Stu |
| *Sorghum bicolor* | XP_002446022.1 | 340 aa | ARG_Sbi |
| *Theobroma cacao* | XP_007019835.1 | 339 aa | arginase isoform 1; ARG1_Tca |
| *Triticum aestivum* | AGR45902.1 | 340 aa | ARG_Tae |
| *Triticum turgidum* | KJ631053 | 340 aa | ARG_Ttu |
| *Triticum urartu* | EMS66394.1 | 340 aa | ARG_Tur |
| *Vitis vinifera* | CBI38896.3 | 371 aa | ARG_Vvi |
| *Zea mays* | ACF85494.1 | 340 aa | ARG_Zma |

*Abbreviation in Fig. 10
